# A Deep Representation Learning Method for Quantitative Immune Defense Function Evaluation and Its Clinical Applications

**DOI:** 10.1002/advs.202515929

**Published:** 2026-01-15

**Authors:** Zhen‐Lin Tan, Tao Luo, Yu Lin, Xiao‐Jun Wu, Wen‐Kang Shen, Jie Chen, Qian Lei, An‐Yuan Guo

**Affiliations:** ^1^ Hubei Key Laboratory of Bioinformatics and Molecular‐imaging College of Life Science and Technology Huazhong University of Science and Technology Wuhan Hubei China; ^2^ Department of Laboratory Medicine West China Biomedical Big Data Center West China Hospital Sichuan University Chengdu China

**Keywords:** deep learning, immune defense, immune score, infectious disease, transcriptome

## Abstract

The immune defense function protecting the body from invasive pathogens is a key indicator of an individual's health and lacks of methods for quantitative evaluation. This study introduces ImmuDef, a novel algorithm for precisely and quantitatively assessing anti‐infection immune defense function based on RNA‐seq data. ImmuDef selects immune signatures through comparisons of acquired immunodeficiency syndrome (AIDS) or severe sepsis vs. healthy controls (HC) and reduces dimension to construct a latent space via a variational autoencoder (VAE) model (QImmuDef‐VAE), a representation deep learning model. Based on this model, a defense immune score (DImmuScore) was calculated by measuring the distance between a patient and HC within latent space. We validated ImmuDef on 3202 samples across four immune states: immunodeficiency, immunocompromised, immunocompetent, and immunoactive. As a result, DImmuScore achieves high classification accuracy (mean accuracy: 71.75%–76.25%) among samples with various immune states and infections. Furthermore, DImmuScore can serve as a metric for infectious disease severity, where its gradient directly quantifies disease severity. As an application, DImmuScore can be a strong prognostic indicator, effectively stratifying mortality/survival in both sepsis and COVID‐19 patients with no symptomatic difference. This framework was validated across five infectious diseases, establishing the first quantitative standard for cross‐disease immune defense assessment.

## Graphical abstract

ImmuDef, a novel algorithm to quantitatively evaluate the anti‐infection immune defense function of an individual based on RNA‐seq data via a variational autoencoder (VAE) model. It is validated on 3200+ samples across four immune states with high accuracy. It can serve as a metric for disease severity and prognosis across pathogenic cohorts. ImmuDef is the first quantitative method for cross‐disease immune defense assessment.

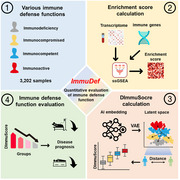

## 1 Introduction

The immune system is an intricate network of cells, tissues, and organs. It protects the body against both external pathogens and internal threats. There are three major functions of the immune system: immune defense, immune surveillance, and immune homeostasis [1, 2]. The immune defense is the system's ability to deploy a coordinated response to combat external infections and other invasions, which mainly includes the ability to against three common types of pathogenic microorganisms: bacterium, virus, and fungus [3]. These microorganism infections are among the most common causes of death in humans [4], and often arise due to a decline in either innate or acquired immunity [5, 6]. Therefore, maintaining robust immune defenses against bacteria, viruses, and fungi is crucial for health.

Individuals with severe acquired immunodeficiency syndrome (AIDS) or in healthy states are considered as two distinct states of immune function: severe immunodeficiency and normal immune function. Compared to these two groups, patients with general microbial infections exhibit immune functions between them [7]. However, their immune systems have not deteriorated to severe immunodeficiency and still retain a significant ability to recover [8, 9], demonstrating an ambiguous immune function state compared to the other two groups. Thus, the immune function of patients with general infections is difficult to measure. Assessing immune function can provide valuable insights into health state evaluation, clinical decisions, disease management, and personalized treatment [10, 11]. Accurate assessment of immune function in infected patients is crucial for timely understanding of their conditions and for guiding clinicians in making appropriate treatment adjustments. However, there is a lack of methods for quantitatively assessing immune defense function.

The conventional rough evaluation of an individual's immune defense function relies on clinical examinations, such as white blood cell counts, cytokine levels, immunoglobulin levels, and inflammatory markers [12, 13]. These are criticized for their lack of precision [14]. Current clinical practice faces three critical gaps in immune monitoring: (a) Existing biomarkers like CD4^+^ counts fail to predict 38% of ART‐associated IRIS mortality until symptom onset [15]; (b) 30% of sepsis deaths occur when sequential organ failure assessment (SOFA) scores show no significant difference (*p* >0.05) between survivors and non‐survivors [16]; (c) Active tuberculosis diagnosis still relies on mycobacterial cultures requiring 2–6 weeks [17]. These limitations underscore the need for a dynamic, quantitative metric of immune defense capacity that can guide preemptive interventions. RNA‐seq data opened a new avenue for understanding immune function, through more personalized and detailed high‐dimensional gene expression information [18]. A study developed the IMM‐AGE metric, which uses longitudinal monitoring of immune parameters to reflect an individual's immune aging state [19]. Ahuja et al. used peripheral blood metrics with gene expression signatures to define optimal immune resilience, which is linked to increased longevity and better prognosis [20]. These methods both rely on multi‐omics data from diverse populations, which makes their application challenging. Clinicians urgently require a single‐time point solution that transcends disease‐specific biomarkers.

In this study, we introduce ImmuDef, a novel framework designed to quantitatively assess immune defense functions. ImmuDef utilizes gene set enrichment analysis (ssGSEA) scores derived from immune‐related gene sets to extract stable immune‐relevant features from the transcriptome. By applying a variational autoencoder (VAE) model, a 2D continuous latent space representation are generated for both qualitative and quantitative immune states assessment [21]. A Defense Immune score (DImmuScore) calculated by ImmuDef was validated across multiple datasets, demonstrating its strong ability to distinguish healthy individuals from those with abnormal immune functions and predict patient prognosis. ImmuDef is a novel method for assessing immune defense function and guiding personalized treatment decisions in clinical situations.

## 2 Results

### 2.1 Overview of ImmuDef

**FIGURE 1:**
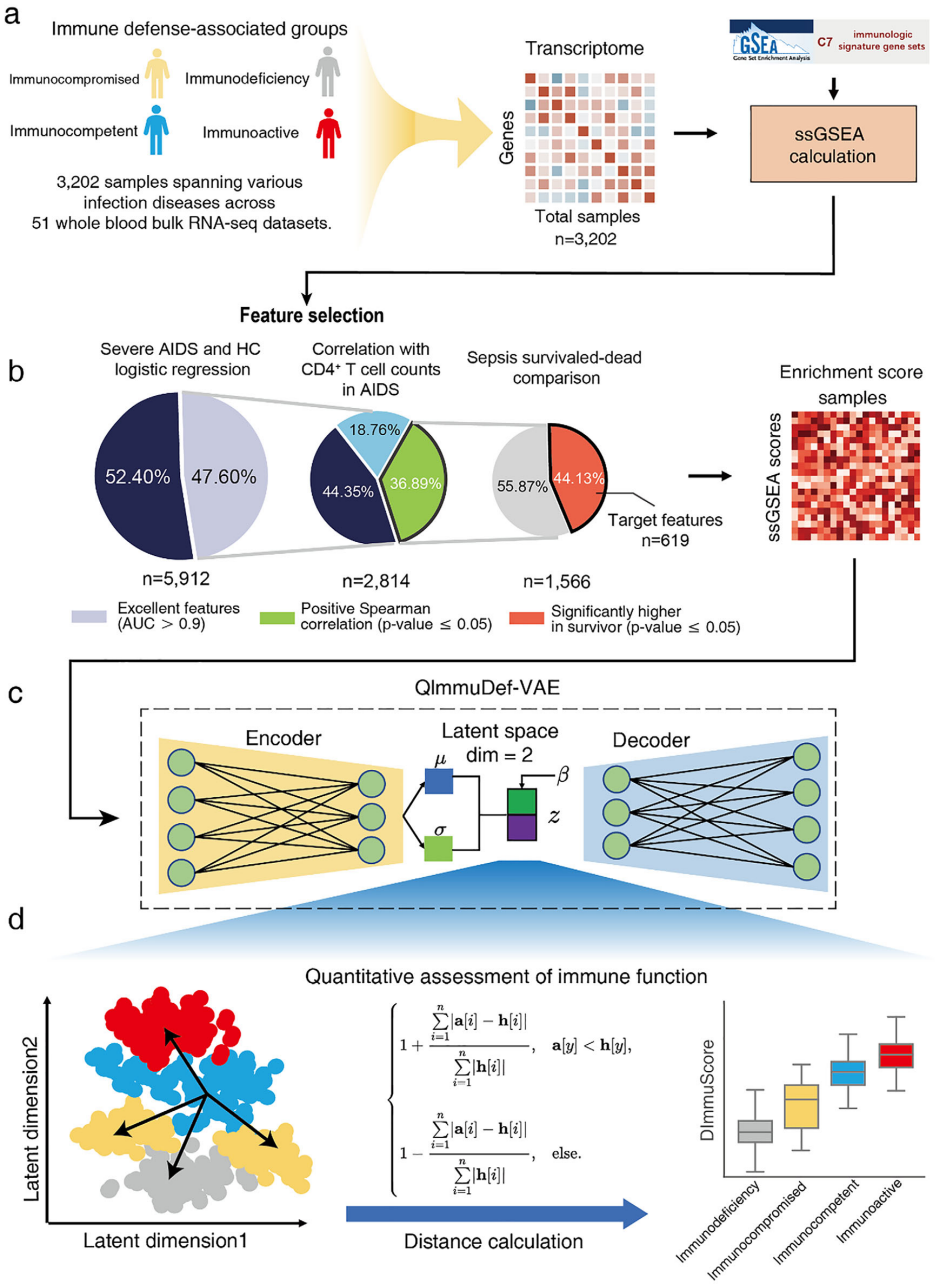
The overview workflow of the ImmuDef algorithm. Overview of ImmuDef: (a) We collected immune defense function‐related transcriptomic data from 3202 patient samples with 4 immune defense function states, and calculated transcriptomic data into ssGSEA score based on MSigDB C7 gene sets. (b) Immune defense‐related ssGSEA scores were selected via a three‐step selection process and 619 features left: (1) Area under curve value (AUC) of the ROC curve ≥0.9 for distinguishing AIDS patients from healthy control (left); (2) Significant positive correlation with CD4^+^ T cell counts in AIDS patients (middle); (3) Significantly enrichment score in sepsis survivors than dead (right). (c) QImmuDef‐VAE model trained on data with features from (b), where the hyperparameter *β* controlled 2D latent space disentanglement. (d) Quantitative analysis of immune defense function under QImmuDef‐VAE embedded latent space.

To address the challenges of quantifying human immune defense function against microbial infections using transcriptome data, we developed ImmuDef, a framework that transforms transcriptomic profiles into stable immune‐relevant features and leverages deep representation learning for robust functional quantification. To improve model robustness and learn functional dynamics in different populations, we collected transcriptome data comprising 3202 samples from various acute/chronic infection patients, and divided them into 4 different immune function states based on their microbial type and pathogenic characteristics (Figure 1a, Supplementary Materials). These data were subsequently converted into ssGSEA scores, which provide enhanced stability by reducing technical variability inherent in count‐based measurements.

To identify clinically significant immune defense features, a rigorous three‐stage filter was applied (Figure 1b, Methods). Features must first distinguish severe AIDS patients from healthy individuals with high accuracy, capturing fundamental immune deficiency pathology [22, 23]. Second, they must positively correlate with CD4+ T cell counts—the gold‐standard biomarker of immune health [24, 25]. Finally, features must show higher levels in surviving sepsis patients, indicating their ability to reflect effective host defense against pathogens [26, 27]. This stringent multi‐condition approach ensured the retained 619 features quantitatively represent core immune defense capacity, linking immune deficiency, functional biomarkers, and survival outcomes—making them vital indicators for downstream predictive modeling of immune defense competence.

To mitigate high‐dimensional complexity, these curated features were processed using a beta‐Variational Autoencoder (VAE) model for quantitative immune defense function (QImmuDef‐VAE) to reduce dimensionality. For computational efficiency and intuitive visualization of immune states across individuals, the latent space was constrained into two dimensions (Figure 1c). This low‐dimensional representation enabled both qualitative stratification of immune function states and quantitative assessment via a novel modified Manhattan distance‐based algorithm. By applying an improved Manhattan distance to measure each sample's latent‐space separation from a rigorously defined healthy reference point—derived from VAE‐generated samples that were rigorously filtered by a consensus ensemble classifier—ImmuDef assigns a continuous score reflecting the extent of immune deviation, thereby enabling quantitative assessment of immune defense capacity (Figure 1d, Methods and Figure S1).

### 2.2 Latent Space Distribution Quantifies Immune Defense Gradient Across Four Functional States

**FIGURE 2:**
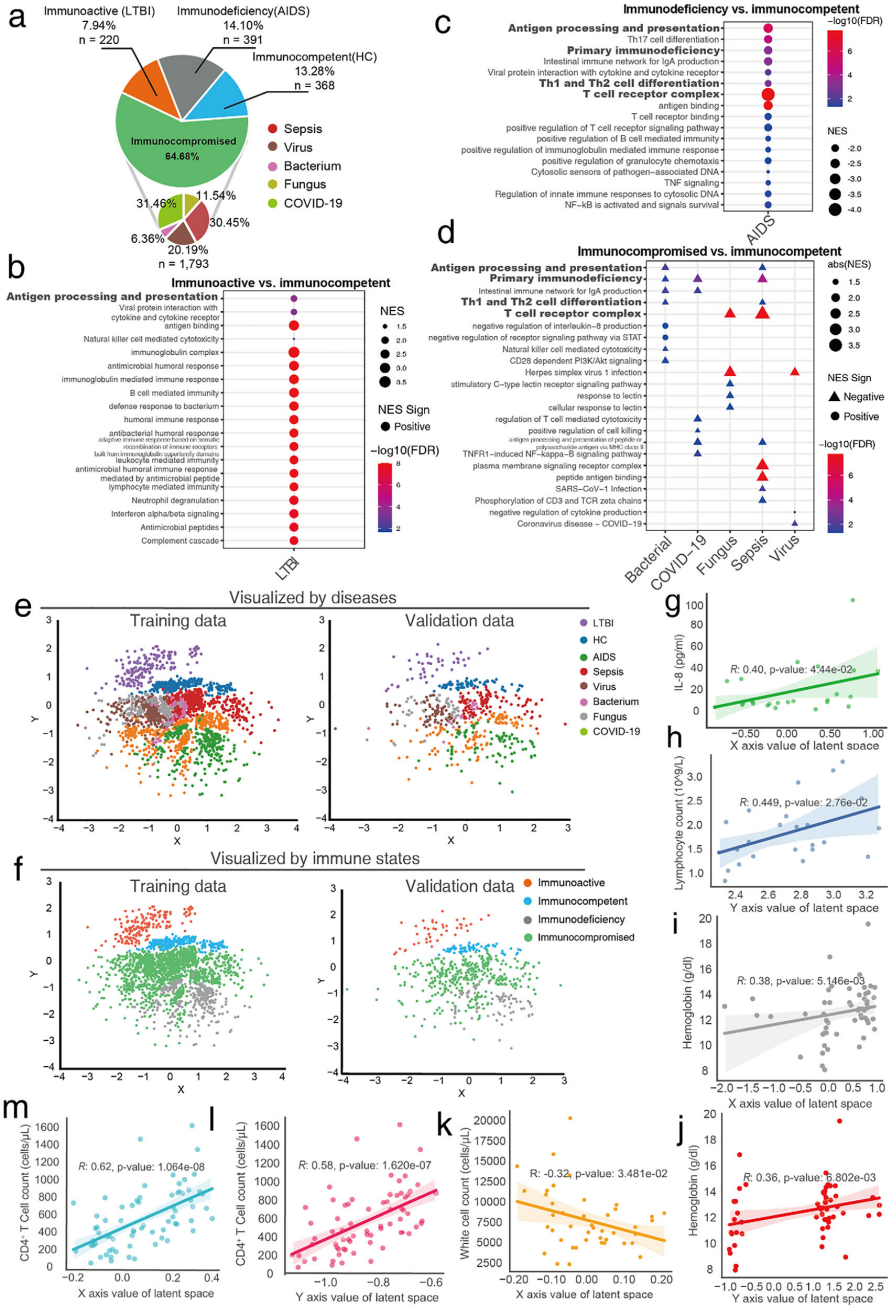
Classification of patients based on immune function states. (a) Composition of the 2816 samples shown in the pie chart (COVID‐19 n = 564, sepsis n = 546, LTBI n = 220, AIDS n = 391, healthy controls n = 368, virus n = 362, fungus n = 207, bacterial n = 114) categorized into 4 immune defense function states (immunoactive n = 220; immunocompetent n = 368; immunocompromised n = 1793; immunodeficiency n = 391). (b–d) GSEA dotplots show significant immune‐related GSEA results calculated by DEGs from (b) immunoactive vs. immunocompetent, (c) immunodeficiency vs. immunocompetent, and (d) immunocompromised vs. immunocompetent (FDR<0.05). (e) Latent space visualization scatter plots with samples stratified by disease categories on (left) training cohort (COVID‐19 n = 452; sepsis n = 436; LTBI n = 220; AIDS n = 312; healthy control n = 294; virus n = 289; fungus n = 165; bacterium n = 91, total n = 2259) and (right) validation cohorts (COVID‐19 n = 112; sepsis n = 110; LTBI n = 44; AIDS n = 79; healthy control n = 74; virus n = 73; fungus n = 42; bacterium n = 23, total n = 557). (f) Latent space visualization scatter plots with samples stratified by immune defense states on (left) training cohort (immunoactive n = 176; immunocompetent n = 294; immunocompromised n = 1434; immunodeficiency n = 313; total n = 2259) and (right) validation cohorts (immunoactive n = 44; immunocompetent n = 74; immunocompromised n = 359; immunodeficiency n = 78, total n = 557). (g) Correlation between hepatitis B patients (n = 25) IL‐8 concentration (pg/ml) and latent space *x*‐axis coordinates (Spearman's R = 0.40, *p*‐value = 4.44 × 10^−2^). (h) Correlation between hepatitis B patients (n = 25) lymphocyte count (10^9^/l) and latent space *y*‐axis coordinates (Spearman's R = 0.45, *p*‐value = 2.76 × 10^−2^). (i,j) Correlation between tuberculosis (n = 54) hemoglobin (g/dl) and latent space coordinates: (i) *x*‐axis (Spearman's R = 0.38, *p*‐value = 5.15 × 10^−3^), (j) *y*‐axis (Spearman's R = 0.36, *p*‐value = 6.80 × 10^−3^). (k) Correlation between AIDS and tuberculosis patients (n = 69), white blood cell count (per microliter), and latent space *x*‐axis coordinates (Spearman's R = −0.32, *p*‐value = 3.49 × 10^−2^). (l,m) Correlation between AIDS patients (n = 69) CD4^+^ T cell counts (per microliter) and latent space coordinates: (l) *y*‐axis (Spearman's R = 0.62, *p*‐value = 1.60 × 10^−8^), (m) *x*‐axis (Spearman's R = 0.58, *p*‐value = 1.62 × 10^−7^). LTBI: Latent Tuberculosis Infection. HC: healthy control. DEG: differentially expressed genes.

After obtaining samples from various infectious patients, we categorized individuals into four distinct immune function states based on their microbial type, pathogenic characteristics, and gene set enrichment analysis (GSEA) results of differentially expressed genes (DEGs) calculated using healthy individuals as a reference (Figure 2a): (1) HIV‐negative latent tuberculosis infection (LTBI) patients were classified as immunoactive, as they effectively control M. tuberculosis over the long term, resulting in either recovery or a non‐contagious latent infection, with intact immunity characterized by activated innate response [28]; and GSEA results revealed that 38 of 43 pathways in LTBI exhibit positive NES values, including NOD‐like receptor signaling, Toll‐like receptor signaling, and cytokine interactions, are highly activated, demonstrating a robust innate and adaptive immune response (Figure 2b). (2) Healthy individuals were classified as immunocompetent, showing no signs of active infection or immune hyperactivation, and maintaining a baseline state of normal immune function. (3) Patients infected with other viral, bacterial, or fungal pathogens (e.g., COVID‐19, Metapneumovirus, Candida spp.) with no HIV infection were classified as immunocompromised, reflecting that these infections generally occur in individuals with weakened immune responses, even in the absence of overt primary immune deficiencies [29, 30]. Furthermore, GSEA results of immunocompromised groups showed that key immune pathways, such as “T cell receptor complex” and “Antigen processing and presentation”, exhibit significant negative enrichment. These down‐regulated pathways, critical for T cell activation and antigen presentation, indicate impaired adaptive immunity across these disease groups (Figure 2d). (4) AIDS patients were classified as immunodeficiency, as their proven profound impairment in immune defense function caused by HIV. Compared to upon groups, AIDS exhibits consistently more significant enrichment score across multiple immune pathways, suggesting that immune function impairment in non‐AIDS groups were generally less severe than in AIDS patients (Figure 2c). Additionally, we performed an unbiased enrichment analysis on the 619 gene sets used for ssGSEA calculation, independent of prior filtering or DEGs information. The results confirmed that a substantial number of these gene sets were significantly enriched in pathways critically involved in immune response and anti‐infection mechanisms (Figures S2, S3, and Table S1).

We examined the distribution of each patient from training data (n = 2259) of QImmuDef‐VAE, a variational autoencoder with a 2D latent space (training benchmarks were detailed in Figure S4). As a result, the latent space distribution of patients could reflect patients’ disease type, in which different diseases have relatively independent distributions (Figure 2e). Especially, the latent space distribution could accurately reflect the four different immune functional states (immunodeficiency, immunocompromised, immunocompetent, and immunoactive) (Figure 2e). Along the *y*‐axis direction, patients were gradually distributed from immunodeficient, immunocompromised, immunocompetent to immunoactive, aligning closely with their immune functional profiles. To further confirm that the latent space captures immune functional dynamics, we applied it to the QImmuDef‐VAE validation dataset (n = 557). Results showed that validation samples exhibited similar spatial distributions as training data (Figure 2f).

Based on the correlation patterns in Figure 2g–m, the two latent dimensions learned by QImmuDef‐VAE capture distinct, biologically meaningful aspects of immune defense capacity. The *y*‐axis exhibits strong positive correlations with markers of overall immune effector strength and host resilience, including CD4+ T‐cell count (Spearman R = 0.62, Figure 2l) in AIDS, lymphocyte count (R = 0.45, Figure 2h) in hepatitis B, and hemoglobin (R = 0.36, Figure 2j) in tuberculosis patients. It also drives the primary separation of immunodeficiency, immunocompetent, and immunoactive states (Figure 2e–f), indicating that it primarily encodes integrated immune effector capacity [31, 32, 33, 34]. In contrast, the *x*‐axis correlates positively with CD4+ T‐cell count (R = 0.58, Figure 2m) in AIDS and hemoglobin (R = 0.38, Figure 2i) in tuberculosis, but also with pro‐inflammatory IL‐8 (R = 0.40, Figure 2g) in hepatitis B, while negatively correlating with total white blood cell count (R = −0.32, Figure 2k) in AIDS. This suggests the *x*‐axis reflects a chronic inflammation and immune maturation–exhaustion axis, with higher values indicating sustained inflammation or T‐cell activation and lower values associated with leukopenia or immune paralysis [31, 32, 34, 35].

Together, these dimensions form an interpretable framework where the *y*‐axis represents the amplitude of immune defense, and the *x*‐axis its quality and sustainability. This biological interpretation underlies the superior prognostic performance of DImmuScore, which assigns greater weight to the *y*‐axis across diverse infections.

### 2.3 Quantitative Assessment of Immune Function by DImmuScore via ImmuDef

**FIGURE 3:**
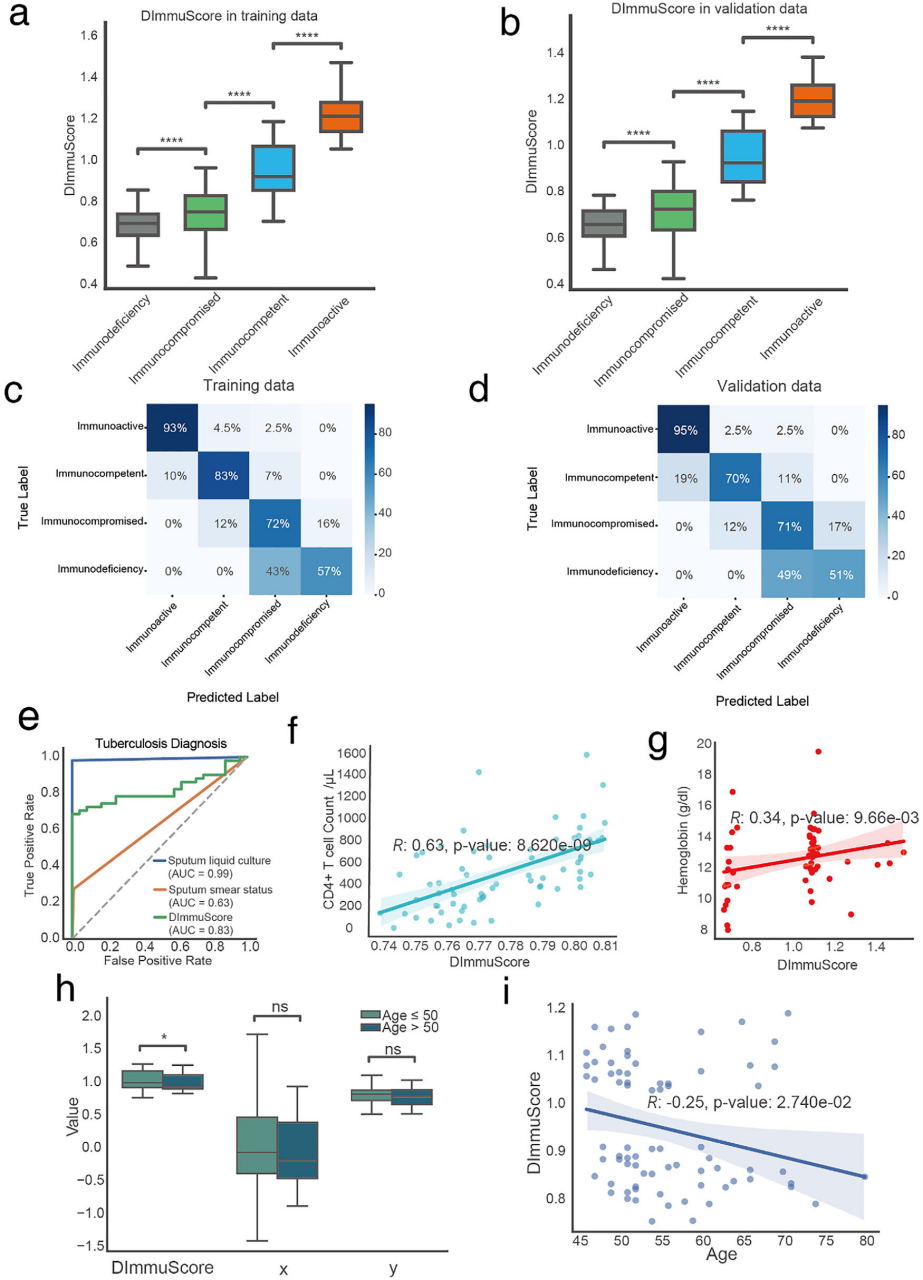
DImmuScore Distribution and Its Classification Performance Among Different Diseases and Immune States. (a,b) Boxplots of DImmuScore distribution stratified by immune function states, on (a) training data (immunoactive n = 176; immunocompetent n = 294; immunocompromised n = 1434; immunodeficiency n = 313; total n = 2,259) and (b) validation data (immunoactive n = 44; immunocompetent n = 74; immunocompromised n = 359; immunodeficiency n = 78, total n = 557). Two‐sided Mann–Whitney U test with Benjamini‐Hochberg correction, ^****^, adjusted *p*‐value ≤ 0.0001. (c,d) Confusion matrices demonstrating DImmuScore's classification results in patients with 4 different immune states of training set (n = 3,077), on (c) training data (immunoactive n = 176; immunocompetent n = 294; immunocompromised n = 1434; immunodeficiency n = 313; total n = 2259) and (d) validation data (immunoactive n = 44; immunocompetent n = 74; immunocompromised n = 359; immunodeficiency n = 78, total n = 557). (e) Comparative diagnostic utility of DImmuScore in TB patients from the PRJNA768419 cohort (n = 75; tuberculosis = 54, no tuberculosis = 21): ROC curves of DImmuScore (AUC = 0.83), gold‐standard sputum culture (AUC = 0.99), and sputum smear (AUC = 0.63) diagnosis performance. (f) Correlation between DImmuScore and CD4^+^ T cell counts (cell counts/microliter) in AIDS patients (n = 69; Spearman's R = 0.63, *p*‐value = 8.62 × 10^−9^). (g) Correlation between DImmuScore and hemoglobin (g/dl) in PRJNA768419 chorots TB patients (n = 54; Spearman's R = 0.34, *p*‐value = 9.66 × 10^−3^). (h) Boxplots of DImmuScore, *y*‐axis values and *x*‐axis values distribution stratified by age (age>50, n = 45; age≤50, n = 248) in immunocompetent/healthy control groups. Two‐sided Mann–Whitney U test, ^*^, *p*‐value ≤ 0.05, ns, no significance. (i) Correlation between DImmuScore and age in immunocompetent/healthy control groups (age≥45, n = 75; Spearman's R = −0.25, *p*‐value = 2.740 × 10^−2^). HC: healthy control. TB: tuberculosis. The significance was assessed by two‐side Mann–Whitney U test, ^****^, *p*‐value ≤0.0001; ^*^, *p*‐value ≤0.05; ns, *p*‐value >0.05. TB: tuberculosis. ns: no significance.

Since the latent space of the QImmuDef‐VAE effectively reflected the continuous variations in immune function across different patients (Figure 2e,f), we proposed DImmuScore—a novel metric calculating the modified Manhattan distance between test samples and the immunocompetent group in the latent space, to quantify immune defense function capacity (Methods). We tested the DImmuScore calculated by ImmuDef on our training data (n = 2259) (Figure 1d). Statistical analysis revealed that DImmuScore robustly distinguished immune functional states: scores for immunodeficiency and immunocompromised group were significantly lower than those of immunocompetent (*p*‐value <1e‐4 across all subgroups), while the immunoactive group exhibited significantly higher scores (*p*‐value <1e‐4), which aligns with their immune function states (Figure 3a). And in the validation dataset (n = 557), the DImmuScore in patients with different immune function groups had consistent performance with the training data (Figure 3b).

To further demonstrate the effectiveness of DImmuScore, we evaluated the classification performance of DImmuScore using confusion matrices from both training and validation datasets (Figure 3c,d). Despite relying on a single feature, DImmuScore achieved outstanding accuracy for the immunoactive class, with 93% and 95% correct predictions in training and validation, respectively, highlighting its strong discriminative power for highly immune‐active profiles. Predictions for immunocompetent and immunocompromised samples also showed robust accuracy, ranging from 70% to 83%, affirming the model's stability across moderately distinct immune states. Notably, we compared alternative embedding methods, including PCA, UMAP, and t‐SNE; although these methods produced visually distinct clusters, their embeddings did not preserve continuous gradients that reflect inter‐patient differences in immune function or have low accuracy in classification across different immune states (Figures S5–S7).

In a cohort of tuberculosis patients from the PRJEB34211 dataset (n = 75; tuberculosis = 54, no tuberculosis = 21) [36]. Comparative diagnostic performance analysis revealed that DImmuScore outperformed sputum smear microscopy (AUC: 0.83 vs. 0.63), despite comparable times (Figure 3e). While sputum liquid culture retained higher diagnostic accuracy (AUC: 0.99 vs. 0.83; requiring 14–42 days for mycobacterial growth), DImmuScore achieved 85% sensitivity with reduction in diagnostic time (Figure 3e).

We tested DImmuScore's correlation with CD4^+^ T cell counts in AIDS patients. The analysis revealed that DImmuScore showed a stronger positive correlation (Figure 3f) than either the *x*‐axis or *y*‐axis of the latent space (Figure 2g,h), which confirmed that DImmuScore effectively quantified immune defense capacity. In addition, a significant correlation was observed between the DImmuScore and peripheral blood hemoglobin levels among tuberculosis patients (Figure 3g).

The physiological age of 40–50 years has been reported in numerous studies as the critical threshold for immune aging, with immune function exhibiting accelerated decline after age 50 [37, 38]. Therefore, we used 50 years as a benchmark to calculate the correlation between DImmuScore and age in immunocompetent/healthy individuals, finding that DImmuScore was significantly lower in individuals over 50 compared to those aged 50 or younger, a result not observed with the original *x*‐axis or *y*‐axis (Figure 3h). Further analysis of DImmuScore's correlation with age revealed a significant negative correlation after age 45, consistent with previous literature reports (Figure 3i).

### 2.4 DImmuScore Reflects Immune States of Patients With Different Disease Severity

**FIGURE 4:**
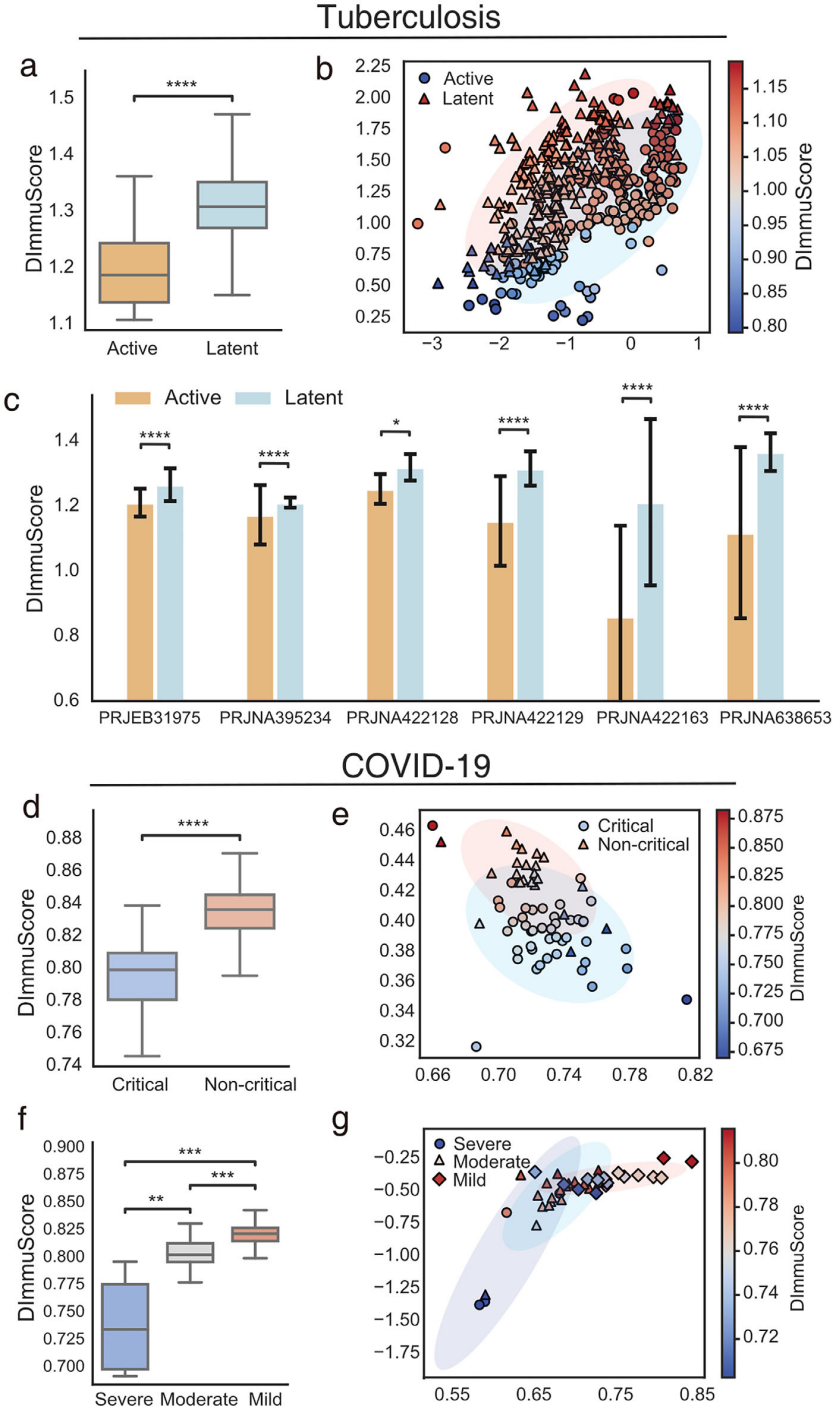
DImmuScore reflects the disease severity in tuberculosis and COVID‐19. (a) Boxplot of DImmuScore distribution in active tuberculosis (n = 222) versus latent tuberculosis (n = 220) cohorts. (b) Scatter plot of latent space representation in tuberculosis patients, with marker color indicating DImmuScore. (c) Bar plot of DImmuScore comparison between active and latent tuberculosis cohorts across multiple datasets. (d) Boxplot of DImmuScore distribution in critical (n = 45) and non‐critical (n = 23) COVID‐19 patients from the PRJNA741686 cohort (total n = 68). (e) Scatter plot of latent space mapping of COVID‐19 patients (n = 68) in different symptoms and colored by DImmuScore. (f) Boxplot of DImmuScore stratification by COVID‐19 severity in the PRJNA722046 dataset (severe n = 4, moderate n = 19, mild n = 19; total n = 40). (g) Scatter plot of latent space representation in COVID‐19 patients from PRJNA722046 dataset, with marker color indicating DImmuScore. The color bar indicates DImmuScore gradient (warmer colors indicate higher values). The significance was assessed by two‐side Mann–Whitney U test, ^****^, *p*‐value ≤0.0001; ^***^, *p*‐value ≤0.01; ^**^, *p*‐value ≤0.01; ^*^, *p*‐value ≤0.05. Scatterplot visualization of data points projected onto the 2D latent space of QImmuDef‐VAE, with axes representing learned latent dimensions. TB: tuberculosis.

After confirming ImmuDef's capacity to stratify immune function states across various diseases, we further evaluated its sensitivity to clinical severity by applying it to patient subgroups stratified by symptom severity. Specifically, we analyzed COVID‐19 and tuberculosis cohorts to assess whether DImmuScore could resolve immune functional differences between mild, moderate, and severe disease states. Figure 4a showed that the DImmuScore in patients with active tuberculosis (n = 222) was significantly lower than in patients with latent tuberculosis (n = 220). Subsequently, we projected the DImmuScore onto the latent space of the tuberculosis patient data and visualized the distribution of tuberculosis patients in latent space (Figure 4b). Latent space visualization revealed distinct distribution patterns between latent and active TB patients, with the magnitude of these spatial differences directly quantified by the DImmuScore and correlated with clinical symptom severity (Figure 4b; Figure S8). Furthermore, we compared the DImmuScore of tuberculosis patients across different Bioproject datasets. The results demonstrated consistent patterns across all Bioproject datasets: patients with latent tuberculosis infection exhibited significantly higher DImmuScore than those with active tuberculosis (Figure 4c). Notably, the immune systems of patients with latent tuberculosis suppress the proliferation of Mycobacterium tuberculosis more effectively than those of patients with active disease states [39, 40].

We analyzed DImmuScore in COVID‐19 patients across severity levels using two datasets: PRJNA741686 (critical n = 45; non‐critical n = 23; Figure 4d) and PRJNA722046 (severe n = 4; moderate n = 19; mild n = 19; Figure 4f). Similar to the tuberculosis patients results, the DImmuScore decreased as the disease severity worsened (Figure 4d,f). Additionally, latent space distributions exhibited significant discriminative capacity between severity groups in both cohorts (Figure 4e,g), and DImmuScore effectively captured these differences in latent space by a single score value (Figure 4e,g; Figure S8).

### 2.5 DImmuScore Prognosticates AIDS Treatment Outcomes

**FIGURE 5:**
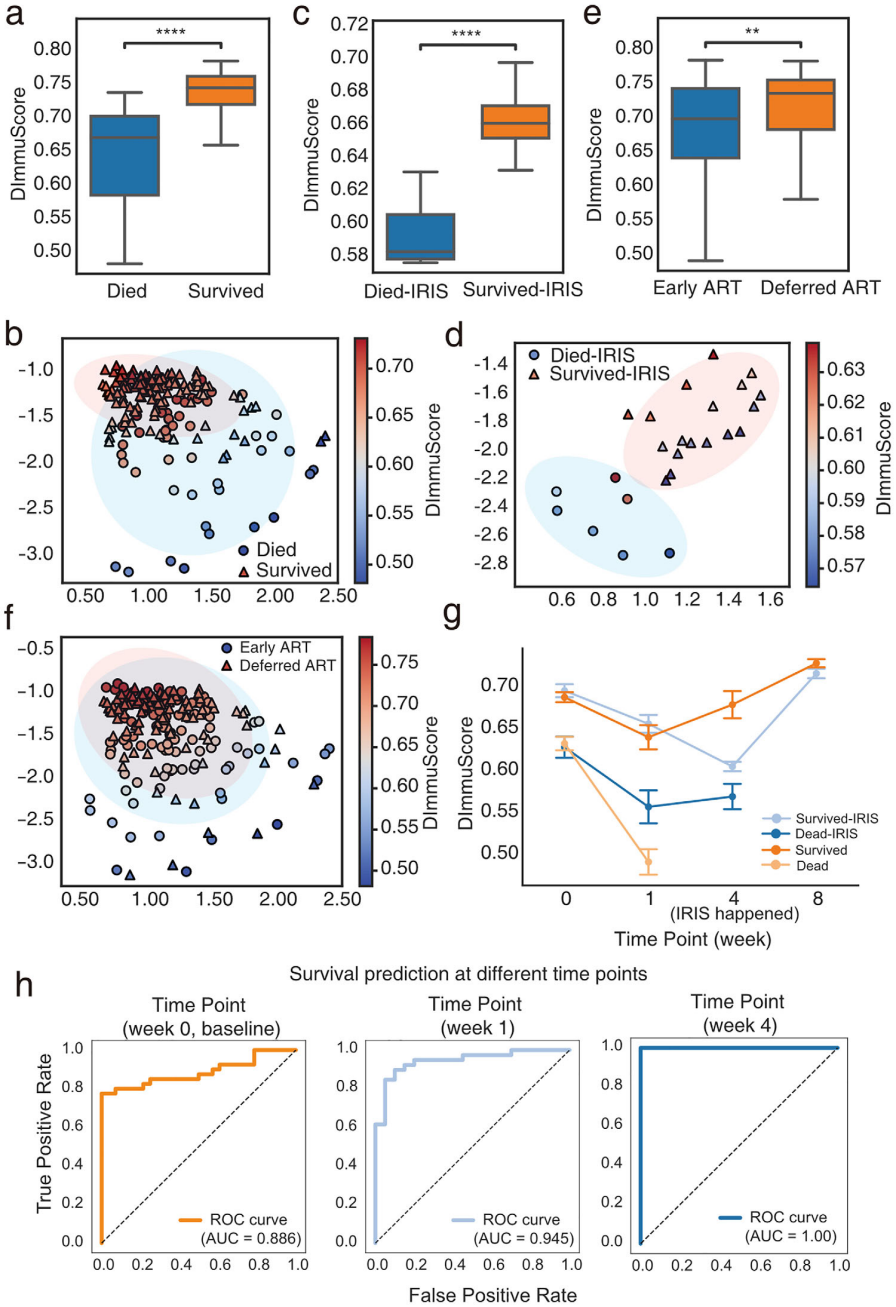
DImmuScore predicts AIDS patient outcome with different treatments. (a,b) Analysis in AIDS patients without IRIS: (a) Boxplot of DImmuScore distribution stratified by survival outcome (died, n = 120; survived, n = 48); (b) Scatter plot of latent space representation (n = 168) with marker color indicating DImmuScore. (c,d) Analysis in AIDS patients with IRIS: (c) Boxplot of DImmuScore in died (n = 7) vs. survived (n = 18) cases; (d) Scatter plot of latent space representation (n = 25) with color‐mapped to DImmuScore. (e,f) Analysis in AIDS patients by ART treatment: (e) Boxplot of DImmuScore distribution stratified by ART treatment (early treatment, n = 102; deferred treatment, n = 91); (f) Scatter plot of latent space representation (n = 193) with color‐mapped to DImmuScore. All above AIDS data were from PRJNA683803 (n = 211) dataset. (g) Longitudinal dynamics of DImmuScore in AIDS patients during treatment from week 0 to week 8. (Dead without IRIS, n = 33; survived without IRIS, n = 70; dead with IRIS, n = 22; survived with IRIS, n = 68). (h) Survival prediction at different time points based on the DImmuScore. Receiver operating characteristic (ROC) curves illustrating the prognostic performance of the DImmuScore at week 0, week 1, and week 4 (dead n = 28, survived n = 40). The area under the curve (AUC) values were 0.886, 0.945, and 1.00, respectively (from left to right). The color bar indicates the DImmuScore gradient. The significance was assessed by two‐side Mann–Whitney U test, ^****^, *p*‐value ≤0.0001; ^**^, *p*‐value ≤0.01; ns, *p*‐value <0.05. Scatterplot visualization of data points projected onto the 2D latent space of QImmuDef‐VAE, with axes representing learned latent dimensions. IRIS: immune reconstitution inflammatory syndrome. ART: antiretroviral therapy. HC: healthy control.

The dynamic changes in immune function of AIDS patients critically influence clinical outcomes, with IRIS (immune reconstitution inflammatory syndrome) a paradoxical inflammatory complication driven by dysregulated CD4^+^ T‐cell recovery, contributing 38% of ART (antiretroviral therapy)‐associated mortality [15]. To investigate this association, we conducted a subgroup analysis using ImmuDef on PRJNA683803 dataset (n = 211), which comprises AIDS patients with cryptococcal meningitis exhibiting diverse clinical outcomes and treatments [41]. Among AIDS patients with/without IRIS, deceased individuals exhibited significantly lower DImmuScore compared to survivors (Figure 5a,b). Furthermore, latent space analysis demonstrated distinct distribution patterns between patients with divergent clinical outcomes (Figure 5c,d). Notably, patients with deferred ART initiation (initiation of ART after 4–6 weeks of antifungal therapy) showed significantly higher DImmuScore than those receiving early ART (initiation of ART within 1–2 weeks of antifungal therapy) (Figure 5e,f) [41]. This observation aligned with the original reporting a 15% survival improvement associated with deferred ART initiation [41].

Our longitudinal analysis of DImmuScore in AIDS patients initiating ART revealed distinct immune recovery trajectories across clinical outcomes. At baseline (week 0), deceased individuals exhibited significantly lower DImmuScore values compared to survivors (Figure 5g; Figure S9). Notably, at week 1 and baseline, DImmuScore already demonstrated predictive capacity for mortality risk, preceding clinical symptom onset by an average of 3–4 weeks (Figure 5g; Figure S9). By week 4, survivors who did not develop IRIS exhibited a marked recovery trajectory, whereas the IRIS‐affected subgroup maintained significantly lower DImmuScore levels. Furthermore, surviving patients overall showed progressive improvement in DImmuScore from week 4 to week 8, aligning with gradual restoration of immune function (Figure 5g). In addition, Figure 5h shows that using DImmuScore at baseline, week 1, and week 4 to predict mortality before clinical deterioration yielded AUC values ranging from 0.886 to 1.0, indicating consistently strong prognostic performance across early timepoints.

### 2.6 Application of ImmuDef in Disease Prognosis Prediction

**FIGURE 6:**
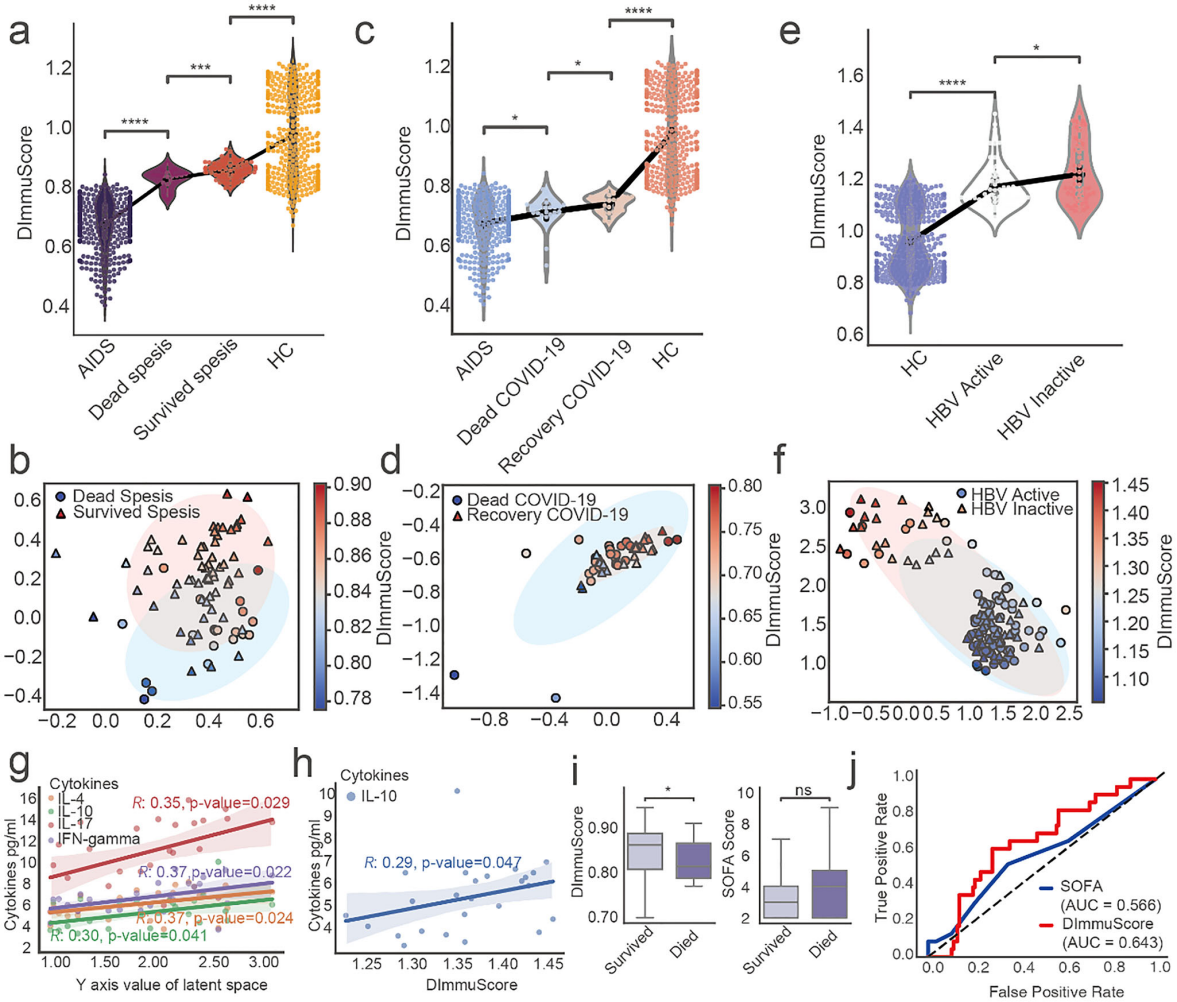
Application of ImmuDef in predicting patient outcomes and patient assessment. (a,b) Analysis in sepsis patients from ICU (BioProject PRJNA768419): (a) Violin plot of DImmuScore distribution stratified by survival outcome (died, n = 20; survived, n = 60); (b) Scatter plot of latent space representation (n = 80). (c‐d) COVID‐19 analysis (PRJNA815981 dataset): (c) Violin plot of DImmuScore distribution stratified by survival outcome (died, n = 24; recovered, n = 24) in patients with matched baseline symptoms (two‐sided Wilcoxon test, *p*>0.05); (d) Scatter plot of latent space representation (n = 48) with color‐mapped to DImmuScore. (e,f) Hepatitis B analysis: (e) Violin plot of DImmuScore distribution stratified by clinical phase (active, n = 73; inactive, n = 52); (f) Scatter plot of latent space representation (n = 125). (g) Correlation analysis between the serum cytokine concentration (pg/ml) of our collected hepatitis B patients (n = 30) and the latent space *y*‐axis. (h) DImmuScore correlation with serum cytokine concentrations (pg/ml) in hepatitis B patients (n = 30). (i,j) DImmuScore diagnosis and predict performance compared with SOFA score in emergency room survived/died sepsis patients (SOFA score≥2) from PRJNA768419 (n = 87; Died = 15, Survived = 72): (i) Significant difference in DImmuScore died‐survived groups (*p*<0.05, left); No significant difference in SOFA scores between died‐survived groups (*p*>0.05, right); (j) ROC of DImmuScore (AUC = 0.643) and SOFA (AUC = 0.566). The significance was assessed by two‐side Mann–Whitney U test, ^****^, *p*‐value ≤ 0.0001; ^***^, *p*‐value ≤ 0.001; ^*^, *p*‐value ≤ 0.05. Scatterplot visualization of data points projected onto the 2D latent space of QImmuDef‐VAE, with axes representing learned latent dimensions. HC: healthy control. HBV: hepatitis B virus. SOFA: sequential organ failure assessment.

The ImmuDef framework was applied to compute DImmuScore across heterogeneous infectious disease cohorts, thereby enabling the evaluation and prediction of prognosis. The extra data from PRJNA768419 dataset of intensive care unit (ICU) sepsis patients (n = 80) were used for analysis. Those patients had been admitted to the ICU for monitoring before been included in the analysis. According to their ends, these patients had been divided into two groups: dead (n = 20) and survived (n = 60) [16]. Moreover, according to SOFA scores reported by the original publication, there was no statistically significant difference in symptoms at the time point of sample collection between the two patient groups (*p*‐value>0.05, Figure S10) [16]. However, our ImmuDef results clearly showed that DImmuScore exhibited statistically significant differences between the two patient groups (*p*‐value ≤0.0001, Figure 6a; Figure S10). The DImmuScore of dead patients were significantly lower than that of surviving patients, and also significantly lower than HC (Figure 6a). Moreover, patients with similar symptoms but different outcomes exhibited differences in their distributions of latent space and DImmuScore (Figure 6b).

We validated ImmuDef in an independent COVID‐19 cohort (PRJNA815981, n = 24), grouping patients into recovered (n = 24) and died (n = 24) outcomes, with all samples collected prior to clinical endpoints [42]. Based on the SOFA provided by the original article, there was no significant difference between the two patient groups in terms of symptom scores at day 0 (*p*‐value >0.05) [42]. The ImmuDef results indicated that DImmuScore was significantly higher in the recovery group than in the dead group (Figure 6c). Similarly, the latent space distribution of patients with similar symptoms but different outcomes showed distinct patterns (Figure 6d). These differences in distribution were also reflected by the DImmuScore. (Figure 6d).

To extend our analysis to chronic infections, we applied the ImmuDef framework to hepatitis B patient datasets. The cohort comprised 73 hepatitis B patients with active hepatitis B virus (HBV) replication and 52 patients with cessation of HBV replication. We combined our sequenced hepatitis B patient data (n = 30) with publicly available hepatitis B patient data (PRJNA727526, n = 95). The results indicated that DImmuScore in hepatitis B patients with active HBV replication were significantly lower than hepatitis B patients with cessation of HBV replication (Figure 6e). Similarly, we observed notable differences in latent space distribution and DImmuScore between the two groups (Figure 6f). Additionally, we explored the correlations between the *y*‐axis value of latent space or DImmuScore with clinical cytokine expression in our hepatitis B patients (n = 30). The *y*‐axis value demonstrated significant correlations with IL‐4, IL‐10, IL‐17, and IFN‐gamma (Figure 6g). Furthermore, the DImmuScore was also correlated with IL‐10 (Figure 6h). Together, these findings underscore that hepatitis B patients with inactive HBV replication exhibit better immune defense and inflammatory regulation compared to those with active HBV replication, further supporting the potential of ImmuDef as a tool for assessing immune function in chronic infections.

We validated the predictive performance of DImmuScore in sepsis prognosis on a previously sepsis cohort (n = 87; Died = 15, Survived = 72, PRJNA768419, SOFA score≥2), which was originally designed for biomarker discovery. All enrolled patients had both SOFA assessments and transcriptomic profiles collected within 24 h of hospital admission and prior to outcome determination [16]. Comparative analysis revealed no statistically significant difference in SOFA scores between deceased and surviving patients (p>0.05, Figure 6i). In contrast, DImmuScore demonstrated marked discrimination between outcome groups (p<0.05, Figure 6i), exhibiting significantly higher sensitivity than SOFA in predicting mortality (AUC: 0.643 vs. 0.563, Figure 6j).

## 3 Discussion

ImmuDef presented a novel framework for quantitative immune defense assessment by integrating transcriptomic data with deep learning‐based feature abstraction. By converting RNA‐seq profiles into ssGSEA scores and leveraging a *β*‐VAE model, our approach overcame the noise and batch‐effect while capturing immune function dynamics. Incorporating a distance‐based calculation in the latent space derived from VAE, with healthy individuals as a reference, ImmuDef creatively addresses the challenge of quantifying and visualizing immune defense functions through the DImmuScore. Its 2‐dim latent space could stratify patients into clinically immune states—immunodeficiency, immunocompromised, and immunoactive—and aligned with biomarkers such as CD4^+^ T cell counts.

Different from traditional machine learning methods, AI‐based ImmunDef provided a structured latent space reflecting continuous immune functional change, enabling differentiation of disease subtypes and prognosis. Considering that large or complex deep learning models such as Transformer, BERT, and other large language models were primarily designed for sequence or vision data analysis, they were deemed unsuitable for our objective of deriving continuous‐level data reflecting immune function from tabular‐structured ssGSEA datasets [43, 44, 45]. Furthermore, traditional generative models like VQ‐VAE, autoencoders, and GAN lacked the ability to produce a standardized distribution in their latent spaces [46, 47]. Consequently, we adopted the β‐VAE framework as the final model for our immune function quantification task.

The derived DImmuScore by ImmuDef bridged qualitative stratification with clinical utility, demonstrating diagnostic accuracy across infections and prognostic value in critical patients from sepsis and COVID‐19 cohorts, even though patients’ symptoms showed no significant difference. The ImmuDef's ability to resolve immune variations was underscored by its performance in chronic infections like hepatitis B and tuberculosis, where latent space distributions and DImmuScore correlated with virus proliferation control and inflammatory cytokine profiles, suggesting ImmuDef captured effector and regulatory immune mechanisms [48]. Specifically, the positive correlations with IFN‐gamma and IL‐4 suggest a stronger antiviral immune response, while the correlation with IL‐10 indicates enhanced immune regulatory functions, potentially mitigating liver damage. The positive correlation with IL‐17 points to a heightened inflammatory response, which is linked to disease activity [33]. By transforming the complex transcriptome into immune metrics, ImmuDef advanced personalized and therapeutic decision‐making [49, 50]. Compared to conventional immune function assessment methods like IMM‐AGE, immune health metric, and sc‐ImmuAging that required long‐time detection processes, multi‐cohort validation, and multi‐omics data integration, ImmuDef required only bulk RNA‐seq data from a single time point [19, 20, 51]. This approach simplified data requirements and improved practicality. Besides, like other methods, DImmuScore also supports comparative analyses across disease subtypes and heterogeneous disease contexts [19, 20, 51].

While the ImmuDef method has shown potential in quantifying immune function and predicting prognosis, our research still has some limitations. And it is essential to note that the method utilizes the ssGSEA score derived from RNA‐seq data, and biases could still be introduced by transforming RNA‐seq data to the ssGSEA score [52, 53]. Additionally, given that the immune system is a typical complex system, our computational method is not capable to comprehensively assess overall immune system [54, 55, 56]. Due to a lack of a large amount of multi‐omics data, such as matched RNA‐seq, TCR‐seq, BCR‐seq, and clinical test results from the same individual of various types of infectious diseases, our method cannot perform a more comprehensive and accurate calculation. Finally, because of the clinical complexity of infectious diseases and the substantial logistical and ethical challenges associated with collecting long‐term, multi‐timepoint patient samples, we were unable to establish a well‐powered prospective cohort for further validating the predictive performance of our model. These elements can be further explored in future studies.

In conclusion, ImmuDef provides a quantitative algorithm for evaluating immune defense functions; it yields infection‐specific outcomes, validated through feature‐discovery and validation datasets. The DImmuScore calculated by ImmuDef can be used to represent the disease severity at various stages of the same infection and predict patient prognosis. Low DImmuScore often indicates poor prognosis or more severe disease states. Clinically, DImmuScore could serve as a clinical indicator to stratify infection severity, monitor disease progression in real time, and predict patient outcomes, thereby informing tailored therapeutic interventions. Additionally, this framework may enhance clinical trial design by enabling patient stratification based on immune function profiles. As our method identifies sepsis at‐risk patients before SOFA score divergence, addresses IRIS mortality in AIDS management through deferred ART timing aligned with immune reconstitution, and replaces mycobacterial cultures with rapid RNA‐seq for tuberculosis diagnosis. In a word, ImmuDef is the first method for quantitative evaluation of immune defense function, which provides guidance for developing methods to calculate other immune functions.

## 4 Materials and Methods

### 4.1 Design of ImmuDef

The concept of the ImmuDef model consists of two major components: (1) the training of QImmuDef‐VAE for its immune defense function representable latent space, and (2) the DImmuScore calculate base on the latent space coordinates, which use healthy control as the reference sample. First, we transformed unstable and high‐dimensional transcriptome data into single‐sample gene enrichment analysis (ssGSEA) data, and selected immune defense function‐related ssGSEA features. Based on these features, we trained a β‐Variational Autoencoder (VAE) model, and set its latent space to *dim*  =  2. At last, the latent space coordinates were calculated to DImmuScore by using the modified Manhattan distance method, which uses healthy control as the reference sample.

### 4.2 Transcriptome Data Collection and Processing

Data of healthy controls, AIDS patients, and subjects with infection‐related diseases were collected through the following process. The retrieval of data involved sourcing SRA or fastq files from NCBI and EMBL‐EBI databases using search terms tailored to each group, including “healthy control whole blood RNA‐seq” and “(disease name) whole blood RNA‐seq”. The samples were required to be whole blood samples from non‐pregnant individuals of the Homo species, categorized as healthy controls or patients with infection‐related or immune‐related diseases.

The alignment of transcriptome data was executed using HISAT2 [57]. The conversion and sorting of SAM to BAM format was completed using Samtools [58]. Following alignment, FeatureCount was employed for the final stage of gene expression quantification, utilizing GENCODE v31 annotations based on the GRCh38.p13 genome annotation file [57, 59].

The main objective of this study was to quantitatively evaluate immune defense functions. To achieve this, we used the C7: immunologic signature gene sets (comprising 5219 gene sets) from the MSigDB as the reference for calculating ssGSEA scores, and the gene expression data were transformed into ssGSEA scores via the Gene Set Variation Analysis (GSVA) package in R [60, 61]. All ssGSEA scores underwent subsequent outlier value correction and standardization. Score points that deviated beyond the median ± 4 standard deviations within the same patient cohort were rectified using the KNNImputer of sklearn, which leverages the k‐nearest neighbors algorithm for data imputation [61]. All processed samples were normalized by the Z‐score method.

### 4.3 Feature Engineering and Immune Defense Quantitative Features Selection Algorithms

To assess immune defense function, we selected gene sets that related to it. Given the significant differences in immune function between healthy controls and AIDS patients, it is essential to identify and prioritize the features that exhibit significant differences between these two groups. Consequently, we deemed single‐feature logistic regression to be an optimal choice for the feature selection task. When comparing healthy control data **H** with severe AIDS data **A**, feature set ***F*** was utilized to classify the two groups:

$$\;l_{p}=\frac{1}{1+e^{-\left(w_{1}\left[H\left[f_{i}],A[f_{i}\right]\right]+w_{0}\right)}}$$

where *f_i_* is the feature *i* in ***F***, [·, ·] represents the concatenation operator, ***l***_***p***_ is the prediction label, *w*_0_ and *w*_1_ are the parameters of the model.

Based on the prediction of AIDS patients and healthy controls data on the completed training model ***l***_***p***_, and the total ground truth labels of healthy controls and AIDS patients of the input data ***l***_***t***_, the immune‐related feature was selected following the algorithm:

Algorithm I **Describes an immune‐related feature selection algorithm.**

**Table:** 

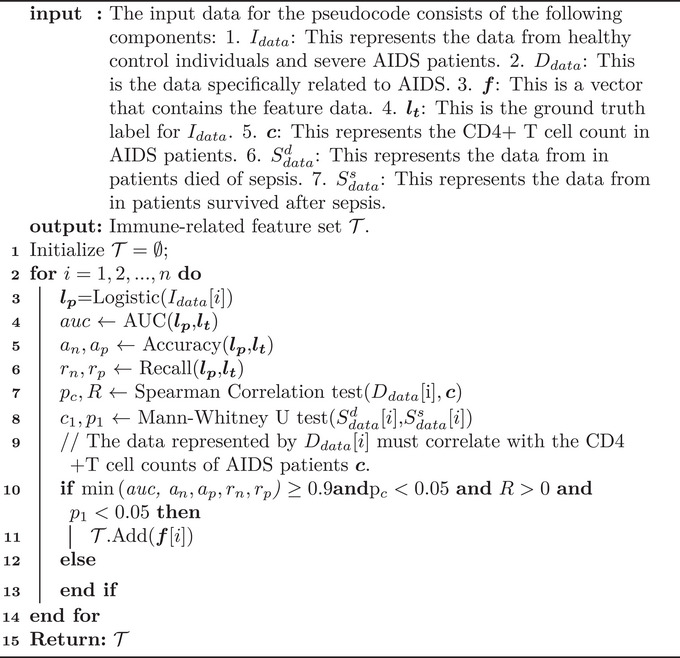

The features that met all the specified criteria with all metrics ≥0.9 and demonstrated a positive correlation with CD4^+^ T‐cells in AIDS patients and exhibited a significant difference in survival‐mortality comparisons of sepsis patients were selected as T, the features that met all metrics were detailed in the Table S2.

### 4.4 QImmuDef‐VAE Training

QImmuDef‐VAE is a variational autoencoder framework composed of two core components: an encoder and a decoder. The model processes ssGSEA data $X\;={\left\{x^{\left(i\right)}\right\}}_{i=1}^{N}$ as input, where each sample $x\;=\{x_{1},\;x_{2}\text{…},\;x_{n}\}$ represents an n‐dimensional feature vector. Each element ***x***_***k***_ in the feature vector, corresponds to the ssGSEA score of the k‐th gene set in T. Through training optimization, aims to reconstruct the original input ***X*** with minimal reconstruction error, preserving the essential patterns in ssGSEA feature, with minimal error while preserving essential patterns in ssGSEA features and constructing latent space representations. The encoder probabilistically maps high‐dimensional inputs ***x*** into a lower‐dimensional latent space ***z*** through parameters ***μ*** and ***σ*** ^2^:

(1) $$q_{\phi }\;\left(z|x\right)=N\left(z;\mu ,\sigma^{2},I\right)$$

where ϕ denotes the encoder's parameters.

The decoder reconstructs  *p*_θ_(***x*** | ***z***) to restore ***x*** from latent variables ***z***, where the θ represents the decoder's parameters. Using variational inference to approximate the true posterior *q*_ϕ_(***z*** | ***x***) and true posterior  *p*_θ_(***z*** | ***x***):

(2) $$\begin{array}{ccc} D_{\mathrm{KL}}\;\left(q_{\phi }\left(z|x\right)\;|\;p_{\theta }\left(z|x\right)\right) & = & \log\left(p_{\theta }\left(x\right)\right)+D_{\mathrm{KL}}\left(q_{\phi }\left(z|x\right)\;|\;p_{\theta }\left(z\right)\right)\\ & & -E_{q_{\phi }\left(z|x\right)}\left[\log\left(p_{\theta }\left(x|z\right)\right)\right] \end{array}$$

where $D_{\mathrm{KL}}$ is Kullback–Leibler divergence.

Rearranging this equation yields:

(3) $$\begin{array}{ccc} \log\left(p_{\theta }\left(x\right)\right)-D_{\mathrm{KL}}\;\left(q_{\phi }\left(z|x\right)\;|\;p_{\theta }\left(z|x\right)\right) & = & E_{q_{\phi }\left(z|x\right)}\;\left[\log\left(p_{\theta }\left(x|z\right)\right)\right]\\ & & -D_{\mathrm{KL}}\left(q_{\phi }\left(z|x\right)\;|\;p_{\theta }\left(z\right)\right) \end{array}$$

The VAE's objective is to maximize log (*p*_θ_(***x***)) or *p*_θ_(***x***) while minimizing $D_{K}\left(q_{\phi }\left(z|x\right)\;|\;p_{\theta }\left(z|x\right)\right)$, equivalent to maximizing the Evidence Lower Bound (ELBO):

(4) $$E_{q_{\phi }\left(z|x\right)}\left[\log\left(p_{\theta }\left(x|z\right)\right)\right]-D_{\mathrm{KL}}\;\left(q_{\phi }\left(z|x\right)\;|\;p_{\theta }\left(z\right)\right)=ELBO$$

In QImmuDef‐VAE, we assume $\;p_{\theta }\left(z\right)\;∼\;N\left(z;\mu ,\sigma^{2I}\right)$, making the KL divergence term $D_{K}\left(q_{\phi }\left(z|x\right)\;|\;p_{\theta }\left(z\right)\right)$:

(5) $$\;L_{KL}=D_{\mathrm{KL}}\left(q_{\phi }\left(z|x\right)\;|\;N\left(z;\mu ,\sigma^{2I}\right)\right)$$

where $L_{KL}$ the loss between *q*_ϕ_(***z*** | ***x***) and Gaussian $N(z;\mu ,\sigma^{2I})$ distribution.

The reconstruction loss is implemented using mean squared error (MSE):

(6) $$L_{Restruction}=MSE\;\left(X|\;\hat{X}\right)=\frac{1}{n}\;\sum_{i=1}^{n}{\left(x_{i}-\hat{x_{i}}\right)}^{2}$$

where **X**, ***x**_i_* denote input samples/features, $\hat{X},\hat{x_{i}}$ denote output samples/features from decoder.

Specifically, a hyperparameter β balances these losses:

(7) $$L=\beta \times L_{KL}+L_{Restruction}$$

The model optimizes parameters θ and ϕ through:

(8) $$min_{\theta ,\phi }L=\beta \times L_{KL}+L_{Restruction}$$

Both encoder and decoder employ multilayer perceptrons (MLPs) where expect the output layer of encoder and decoder, with ReLU activations for all hidden layers. The encoder architecture (619→512→128→32→2 neurons) mirrors the decoder's inverted structure (2→32→128→512→619 neurons), where 619 corresponds to the ssGSEA feature dimension, and we set β  =  0.1 to get an optimized model. We split 80% samples for model training and 20% samples for validation, which by setting random seed 42. Our model was trained on NVIDIA RTX 4090 (24G) for 20 000 epochs (Figure S4), built by Pytorch version (2.0.1) with CUDA 11.8 environment (https://pytorch.org/?pStoreID = Http).

### 4.5 Formation of Reference Healthy Samples

To obtain an optimal healthy reference sample for DImmuScore calculation, we first trained an ensemble learning model composed of SVM, XGBoost, and logistic regression classifiers using ssGSEA scores as input features. Each classifier was independently optimized via grid search and trained on the same QImmuDef‐VAE–derived training dataset. Stratified splitting of the ssGSEA dataset (60% training, 40% validation) with a fixed random seed (42) ensured reproducibility. Leveraging the generative capacity of QImmuDef‐VAE, we uniformly sampled 1,000,000 points across a dense 2D latent grid (x, y ∈ [–4, 4]) and generated the corresponding decoded samples. The ensemble classifier was then applied to these samples to identify high‐confidence healthy‐like profiles. Only samples unanimously predicted as “healthy” by all sub‐models with a probability > 0.9 were retained. The median latent coordinates (x, y) of this filtered subset were taken as the reference point ***h*** for calculating the DImmuScore (see Figure S11).

### 4.6 DImmuScore Calculation

Given that the immune function of all populations, except for healthy controls, is compromised, the final quantification of immune function will be calculated using healthy samples as a reference, which is in QImmuDef‐VAE's embedding latent space and latent variable. The DImmuScore was calculated by referencing the healthy human centroid sample ***h*** embedded by QImmuDef‐VAE, on a modified Manhattan distance method:

$$DImmuScore=\left\{\begin{array}{cc} 1+\frac{\sum_{i=1}^{n}\left|a\left[i\right]-h\left[i\right]\right|}{\sum_{i=1}^{n}\left|h\left[i\right]\right|}, & a\left[y\right]<h\left[y\right],\\ 1-\frac{\sum_{i=1}^{n}\left|a\left[i\right]-h\left[i\right]\right|}{\sum_{i=1}^{n}\left|h\left[i\right]\right|}, & else. \end{array}\right.$$

where ***a*** is the sample which needs to be calculated, *y* is *y*‐axis value of the latent space.

The construction of the reference sample ***h*** construction comprises two‐stage sequential steps: (1) Utilizing a previous ensemble learning model, we computed classification confidence scores for “Healthy Control” membership in the latent space. Samples were retained only if their mean classification confidence across ensemble components>0.95 across all sub‐models; (2) Spatial boundary constraints were applied within the distribution range of healthy control samples from the training dataset, ensuring generated samples reside within known control group regions, with the median of processed coordinates ultimately serving as the reference benchmark.

### 4.7 Classify Patient Groups by DImmuScore

Here was a description of Area Under the Curve (AUC) values for results of our purely DImmuScore numerical classification: In a dataset where samples were grouped into two categories, the objective was to compare two specific groups in order to identify the optimal cut‐off point that distinguished them based on DImmuScore. This process began by identifying the minimum and maximum values of the DImmuScore within the two groups of interest. These values served to define the range for potential optimal thresholds. For each threshold, the samples were classified as either positive or negative based on whether their score was above or below the threshold. With these classifications, a Receiver Operating Characteristic (ROC) curve was constructed by calculating the true positive rate (sensitivity) and false positive rate (1‐specificity) at each threshold. The AUC value, derived from the ROC curve, provided a single scalar value that summarized the performance of the classifier.

### 4.8 Data and Code Availability

All datasets used in this study are publicly available. The details of the datasets used in this study are further provided in Tables S3 and S4. All other clinical data supporting the findings of this study were available within the original articles or their supplementary files. Any additional requests for information can be directed to and will be fulfilled by the corresponding author. The source code for ImmuDef and all results were freely available at https://github.com/GuoBioinfoLab/ImmuDef.

### 4.9 RNA Isolation and Library Preparation for Our Sequenced Hepatitis B Patient Samples

We collected 30 samples of hepatitis B patients from West China Hospital, Sichuan University. Ficoll was used to separate Peripheral blood mononuclear cells (PBMC) from 2 mL of whole blood according to the user manual. The total RNA was extracted using the TRIzol reagent according to the manufacturer's protocol. RNA purity and quantification were evaluated using the NanoDrop 2000 spectrophotometer (Thermo Scientific, USA). RNA integrity was assessed using the Agilent 2100 Bioanalyzer (Agilent Technologies, Santa Clara, CA, USA). Then the libraries were constructed using TruSeq Stranded mRNA LT Sample Prep Kit (Illumina, San Diego, CA, USA) according to the manufacturer's instructions. The transcriptome sequencing and analysis were conducted by OE Biotech Co., Ltd. (Shanghai, China). The libraries were sequenced on an Illumina HiSeq X Ten platform, and 150 bp paired‐end reads were generated. And the criterion for active HBV replication had been defined as an HBV‐DNA test result in peripheral serum <200 IU/ml. The detailed clinical information of hepatitis B patient samples in this study are further provided in Tables S5 and S6.

### 4.10 Differential Expression Genes and GSEA Analysis

The gene expression data were normalized by TMM method in the edgeR package, and genes with consistently low or zero counts were filtered out [62]. Differentially expressed genes (DEGs) were then identified by a Wilcoxon rank‐sum test and Deseq2 [63]. Given the multitude of tests conducted, the resulting *p*‐values were adjusted using the FDR (false discovery rate) method to mitigate the issue of multiple comparisons to avoid false positives. To rigorously remove low‑expression features and further standardize the transcriptome data, raw read counts were converted to Transcripts Per Kilobase per Million (TPM). Only genes with an adjusted p‑value (FDR) ≤ 0.05 and |log_2_ fold change| ≥ 1in at least one comparison group were considered as DEGs by clusterprofiler [64]. For cross‐dataset DEG analysis (integrating patients from multiple studies into disease‐specific cohorts), batch effects were corrected using the ComBat algorithm prior to differential expression analysis, as previously described [65].

After finishing the differentially expressed gene analysis, we further analyzed gene function. We performed gene set enrichment analysis (GSEA) based on gene sets from Gene Ontology (GO, n = 10 447), Kyoto Encyclopedia of Genes and Genomes (KEGG, n = 186), and Reactome (n = 1615) function‐annotated gene sets. We calculated GSEA results based on DEGs identified in the previous step. To ensure the accuracy of the enrichment results, only those gene sets with an adjusted p‑value (FDR) ≤ 0.05 were considered significant.

### 4.11 Statistical Analysis

All statistical analyses were performed using Python 3.9+ with the SciPy (scipy.stats) package. Between‐group comparisons (two groups) were conducted using the two‐sided Mann‐Whitney U test. When multiple hypothesis testing was performed, *p*‐values were corrected for false discovery rate using the Benjamini–Hochberg (BH) procedure. Correlations were assessed by Spearman's rank correlation coefficient. Statistical significance was defined as *p* <0.05 after correction, where applicable. All tests were implemented with default parameters unless otherwise stated. Sample sizes (n) for each group or condition are explicitly reported in the corresponding figure captions. Data preprocessing steps—including feature normalization, transformation, and outlier evaluation—were described in detail in the previous sections.

## Funding

Financial support was provided by the National Natural Science Foundation of China (92574106, 32525021, and 82271599), the 1.3.5 project for disciplines of excellence from West China Hospital of Sichuan University (ZYYC23007), and the Fundamental Research Funds for the Central Universities (YJ202504). The computations in this paper were supported by the High Performance Computing Platform at West China Biomedical Big Data Center, West China Hospital, Sichuan University.

## Ethics Statement

This study was approved by the Ethics Committee on Biomedical Research, West China Hospital of Sichuan University (Reference: HX‐IRB‐AF‐21‐V3.0). Written informed consent was obtained from all human tissue donors prior to sample collection. All donor information was anonymized during data analysis to ensure participant confidentiality.

## Conflicts of Interest

The authors declare no conflicts of interest.

## Supporting information




**Supporting File 1**: advs73806‐sup‐0001‐SuppMat.docx.

**Supporting File 2**: advs73806‐sup‐0002‐Supplementary Tables Revised.xlsx.

## Data Availability

The data that support the findings of this study are available in the supplementary material of this article.
