# Supplementary material for: A Deep Representation Learning Method for Quantitative Immune Defense Function Evaluation and Its Clinical Applications
Source: Adv Sci (Weinh). 2026 Jan 15;13(17):e15929. doi: 10.1002/advs.202515929 (PMC13042766; doi:10.1002/advs.202515929)
Supplement: Supplementary file 1 — Supporting File 1: advs73806‐sup‐0001‐SuppMat.docx. [file ADVS-13-e15929-s002.docx]

## **Supplementary Material**

**Supplementary Figures**

**Supplementary Fig. 1** Feature selection strategy for identifying immune defense signatures using ssGSEA across KEGG, GO and Reactome pathway databases

**Supplementary Fig. 2** GO-based immune functional enrichment of the 619 gene sets used in ImmuDef

**Supplementary Fig. 3** Reactome-based immune functional enrichment of the 619 gene sets used in ImmuDef.

**Supplementary Fig. 4** Training progression of QImmuDef-VAE variational autoencoder with dual-loss monitoring

**Supplementary Fig. 5** Dimensionality-reduction visualization of ssGSEA-derived immune features.

**Supplementary Fig. 6** Boxplots of PCA-based DImmuScore distribution

**Supplementary Fig. 7** Confusion matrices demonstrating PCA-based DImmuScore

**Supplementary Fig. 8** ROC curves and their AUC values for classification results of DImmuScore among different subgroups of tuberculosis and COVID-19 patient datasets

**Supplementary Fig. 9** DImmuScore dynamics in AIDS patients across treatment timepoint

**Supplementary Fig. 10** DImmuScore diagnosis and predict performance compared with SOFA score in ICU survived/died sepsis patients from PRJNA768419

**Supplementary Fig. 11** Heatmaps of predicted average probabilities for nine target classes

**Supplementary Tables**

**Supplementary Table S1.** Immune functional enrichment of the 619 gene sets used in ImmuDef

**Supplementary Table S2.** ssGSEA feature for QImmuDef-VAE training

**Supplementary Table S3.** Gender, age and group information of disease groups

**Supplementary Table S4.** Projects and subgroups of RNA-seq datasets for different diseases

**Supplementary Table S5.** Clinical information of our hepatitis B patients

**Supplementary Table S6.** Patient composition of total data


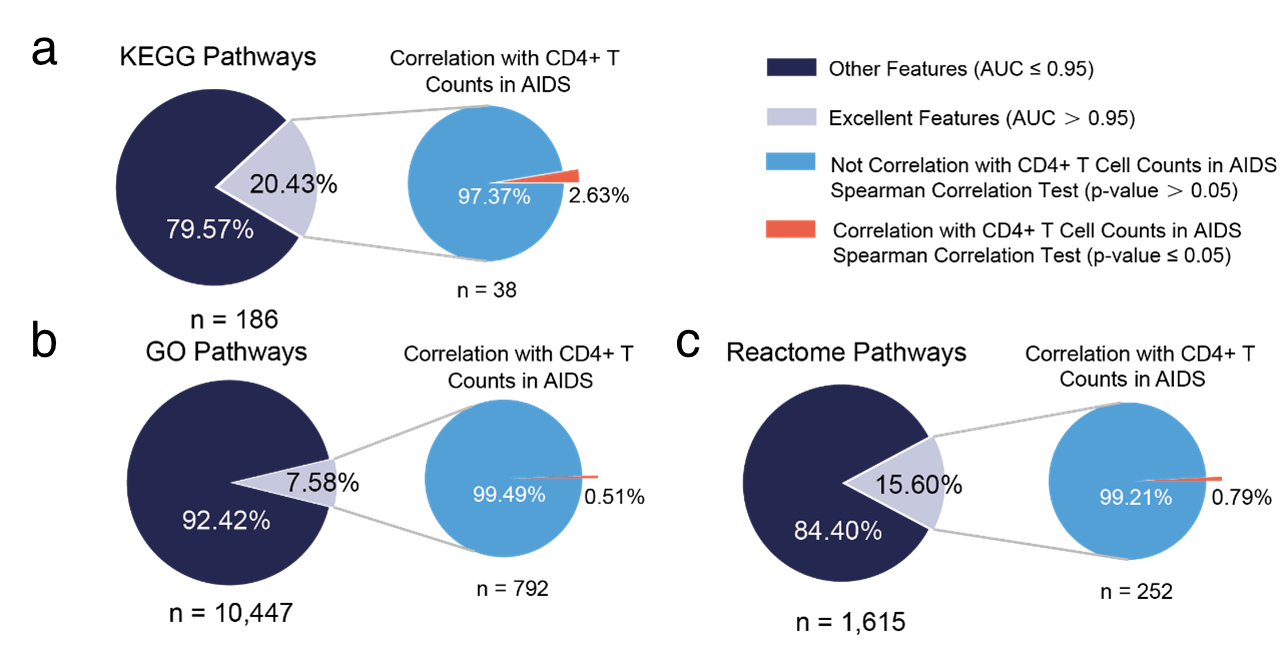


**Supplementary Fig. 1** Feature selection strategy for identifying immune defense signatures using ssGSEA across KEGG, GO and Reactome pathway databases.

ssGSEA scores of immune defense-related features were selected through three sequential criteria: (1) Area under the ROC curve (AUC) ≥ 0.95 for distinguishing AIDS (n=391) patients from healthy controls; (2) Significant positive correlation with CD4+ T cell counts in AIDS patients (Spearman's, n=69); (3) Significantly enrichment score in sepsis survivors than dead (Mann-Whitney U test), through (**a**) KEGG (n=186), (**b**) GO (n=10,447) and (**c**) Reactome (n=1,615) pathway sets. The significance was assessed by two-side Mann-Whitney U test. GO: Gene Ontology. KEGG: Kyoto Encyclopedia of Genes and Genomes.


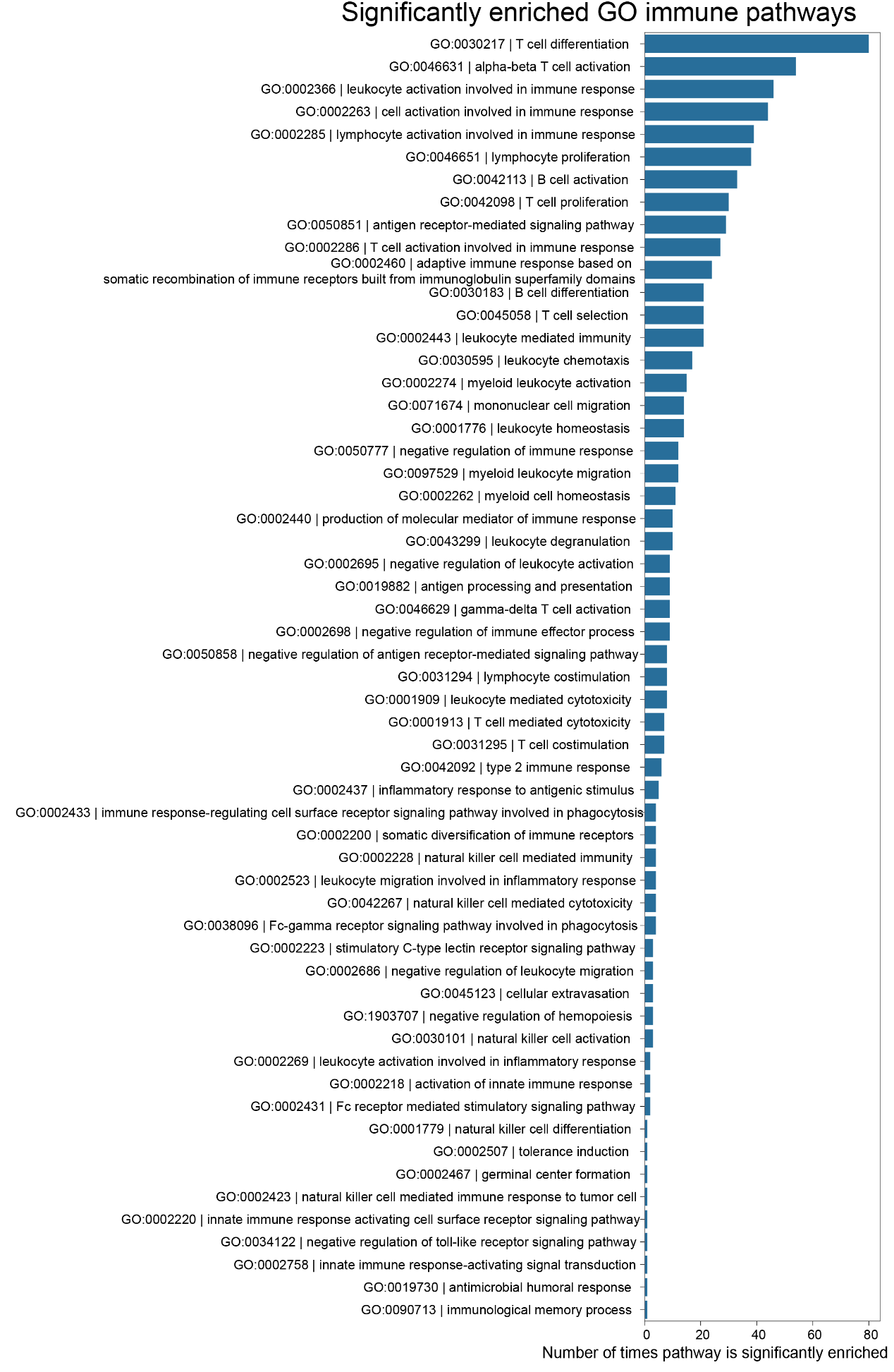


**Supplementary Fig. 2** GO-based immune functional enrichment of the 619 gene sets used in ImmuDef. Immune-related pathways were obtained from the ImmPort database (https://www.immport.org/shared/genelists), and enrichment significance was defined as adjusted p-value < 0.05.


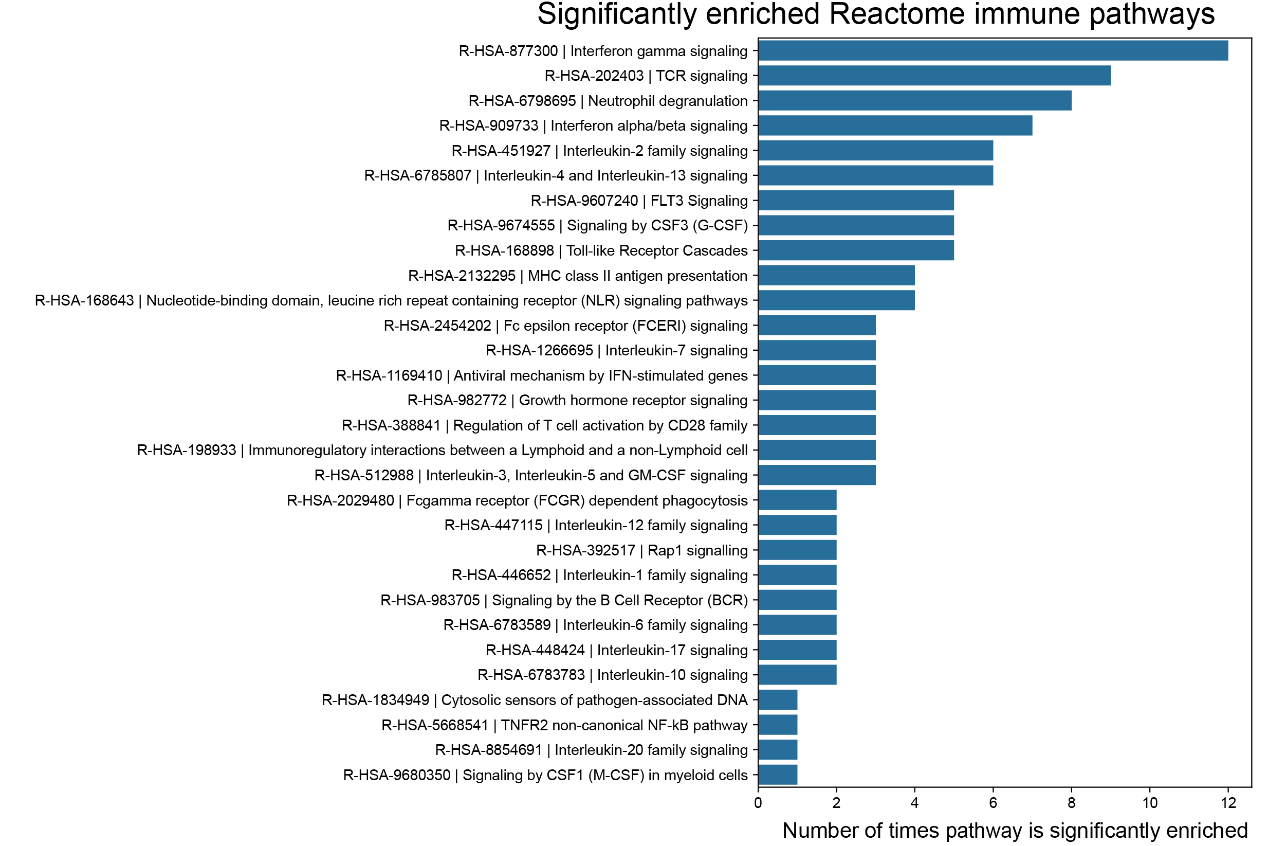


**Supplementary Fig. 3** Reactome-based immune functional enrichment of the 619 gene sets used in ImmuDef. Immune-related pathways were obtained from the ImmPort database (https://www.immport.org/shared/genelists), and enrichment significance was defined as adjusted p-value/FDR < 0.05.


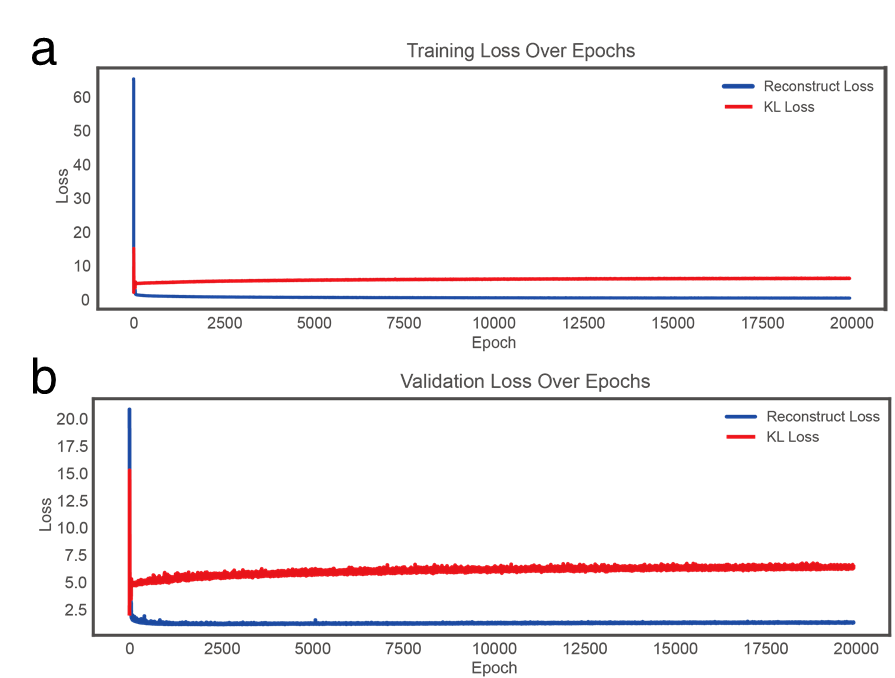


**Supplementary Fig. 4** Training progression of QImmuDef-VAE variational autoencoder with dual-loss monitoring

(**a**) Training trajectory on the discovery cohort (n=2,561 samples), (**b**) Validation performance on the independent hold-out set (n=641 samples). Both panels simultaneously track: KL loss (Kullback-Leibler divergence) and reconstruction loss.


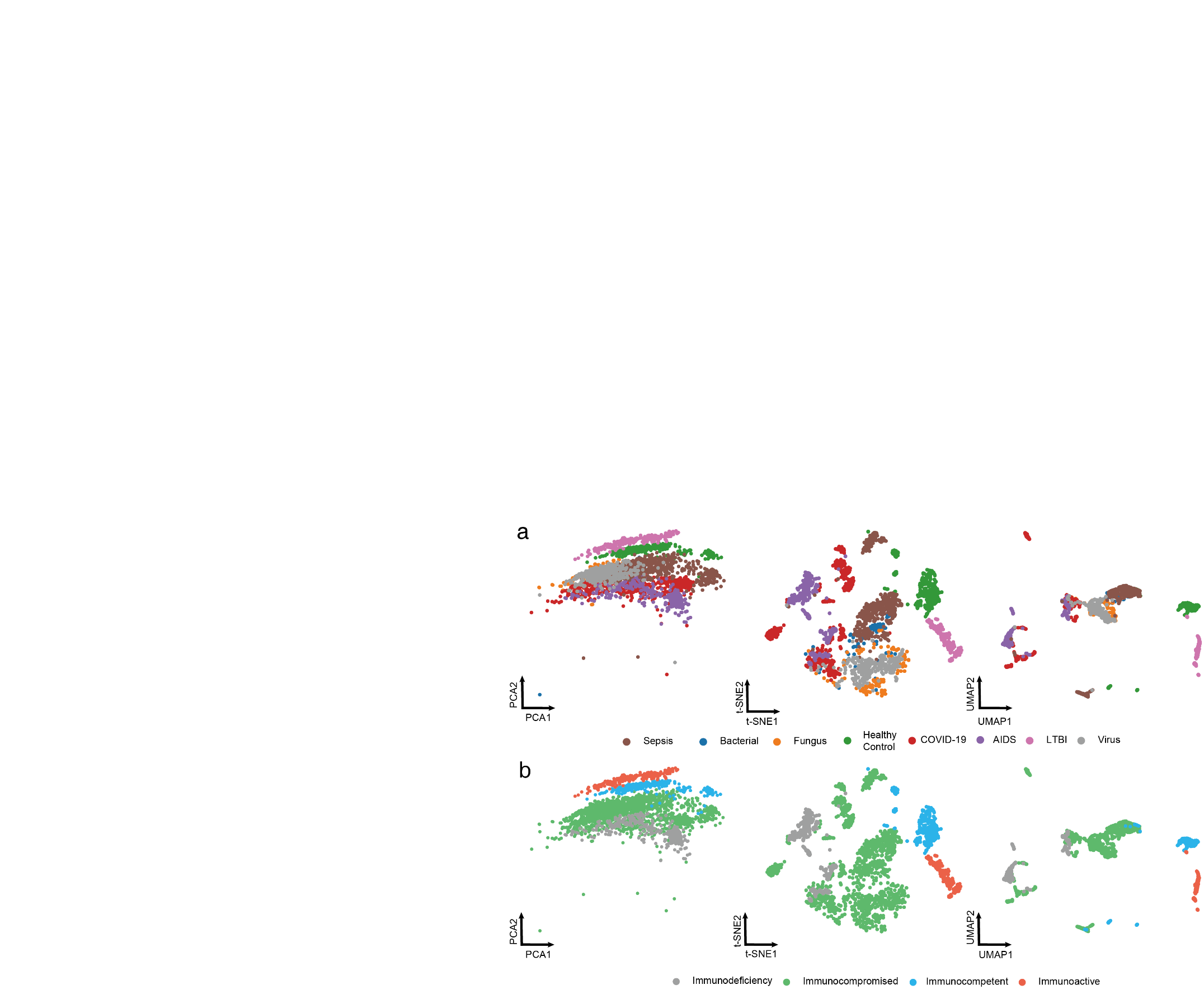


**Supplementary Fig. 5**: Scatter plots of dimensionality reduction results (left to right: VAE, PCA, t-SNE, UMAP) applied to total patient cohorts (immunoactive n=176; immunocompetent n=294; immunocompromised n=1434; immunodeficiency n=313; total n= 2,259), stratified and colored by (a) disease categories and (b) immune defense states.


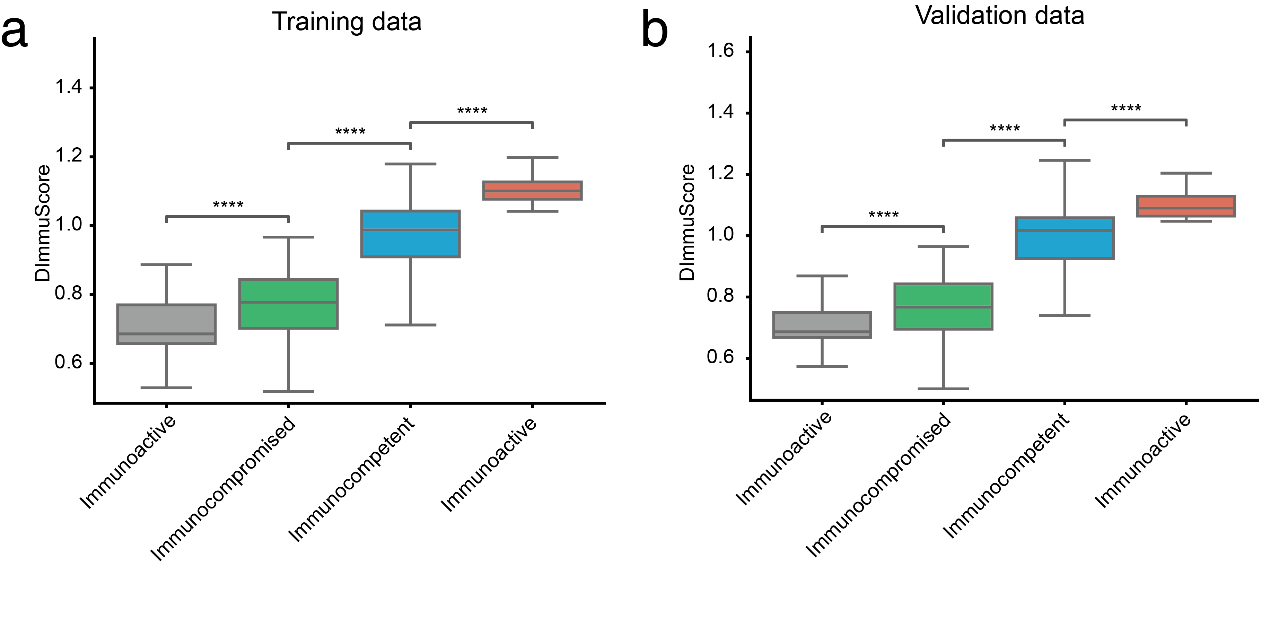


**Supplementary Fig. 6** Boxplots of PCA-based DImmuScore distribution stratified by immune function states, on (a) training data (immunoactive n=176; immunocompetent n=294; immunocompromised n=1434; immunodeficiency n=313; total n= 2,259) and (b) validation data (immunoactive n=44; immunocompetent n=74; immunocompromised n=359; immunodeficiency n=78, total n=557). Two-side Mann-Whitney U test with Benjamini-Hochberg correction, ****, adjusted p-value ≤ 0.0001.


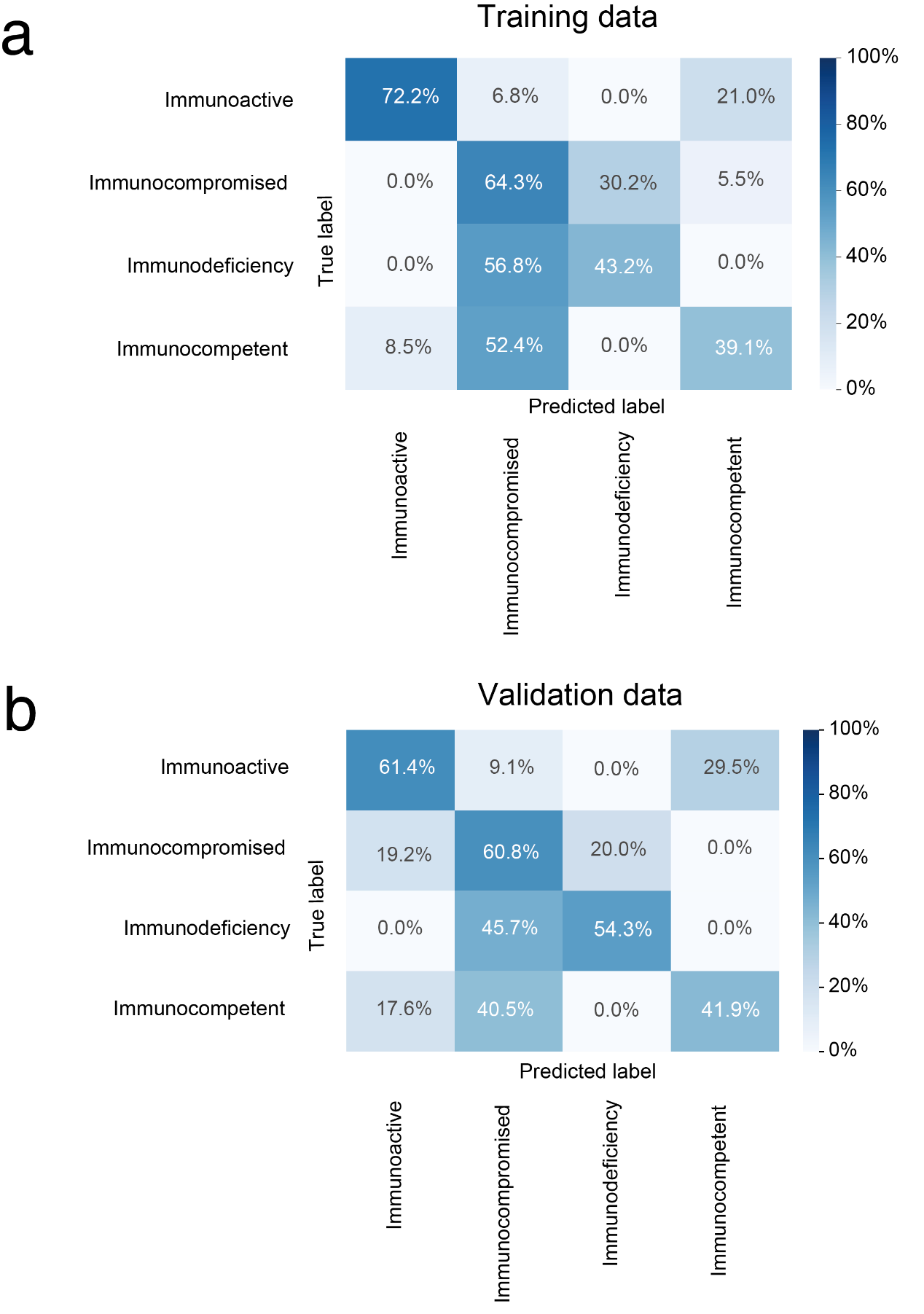


**Supplementary Fig. 7** (a-b) Confusion matrixes demonstrating PCA-based score's classification results in patients with 4 different immune states of training cohort (immunoactive n=176; immunocompetent n=294; immunocompromised n=1434; immunodeficiency n=313; total n= 2,259) and (b) validation cohort (immunoactive n=44; immunocompetent n=74; immunocompromised n=359; immunodeficiency n=78, total n=557).


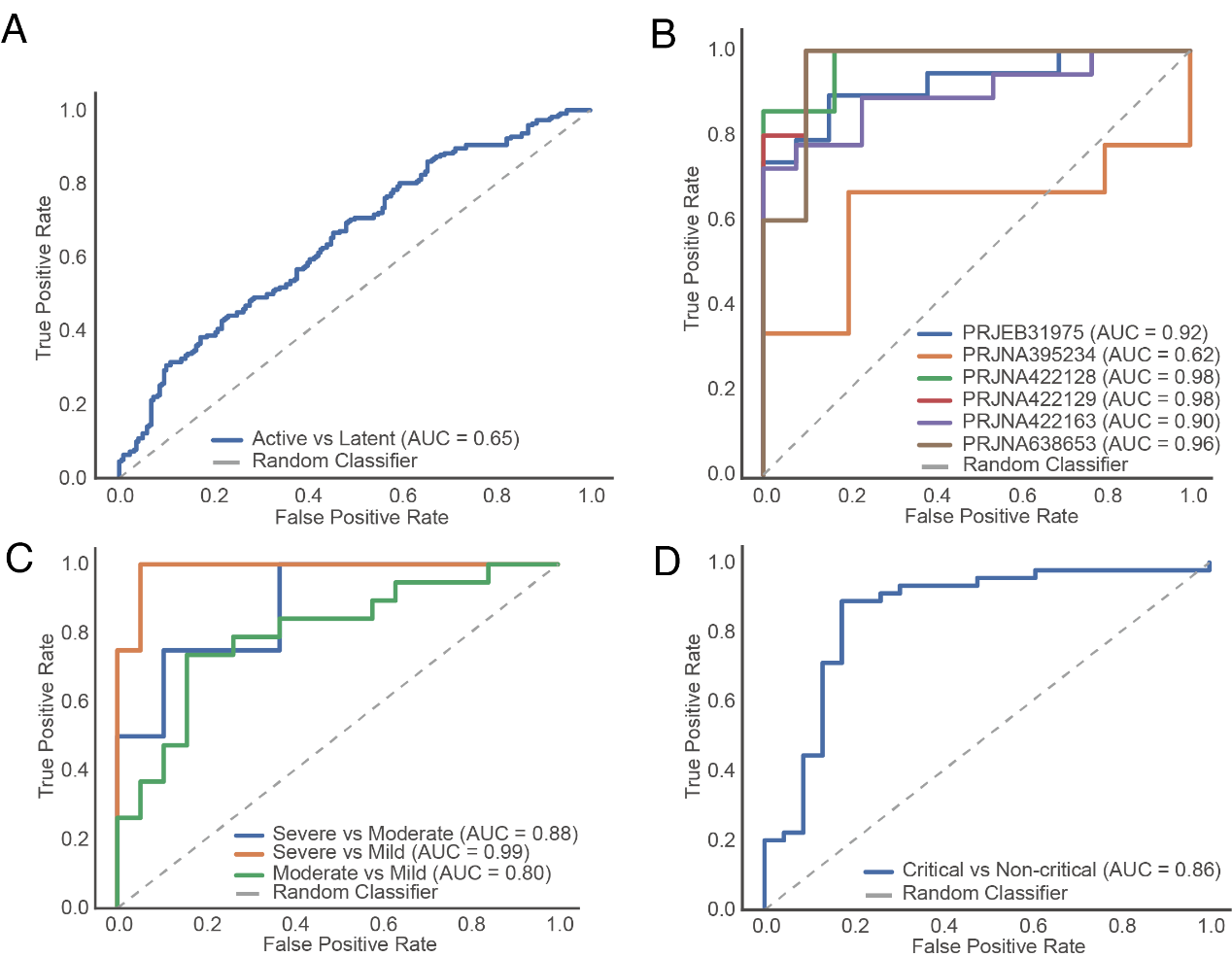
**Supplementary Fig. 8** ROC curves and their AUC values for classification results of DImmuScore among different subgroups of tuberculosis and COVID-19 patient datasets (**a**–**b**) DImmuScore classification performance in tuberculosis patients with different severity: (**a**) in active (n=222) vs. latent (n=220) tuberculosis in total cohorts; (**b**) in Bioprojects PRJEB31975, PRJNA395234, PRJNA422128, PRJNA422129, PRJNA422163, and PRJNA638653 (latent vs. active). (**c**–**d**) DImmuScore COVID-19 classification in patients with different severity: (**c**) PRJNA741686 (severe n=4, moderate n=19, mild n=19; total n=40); (**d**) PRJNA722046 critical (n=45) vs. non-critical (n=23) patients.


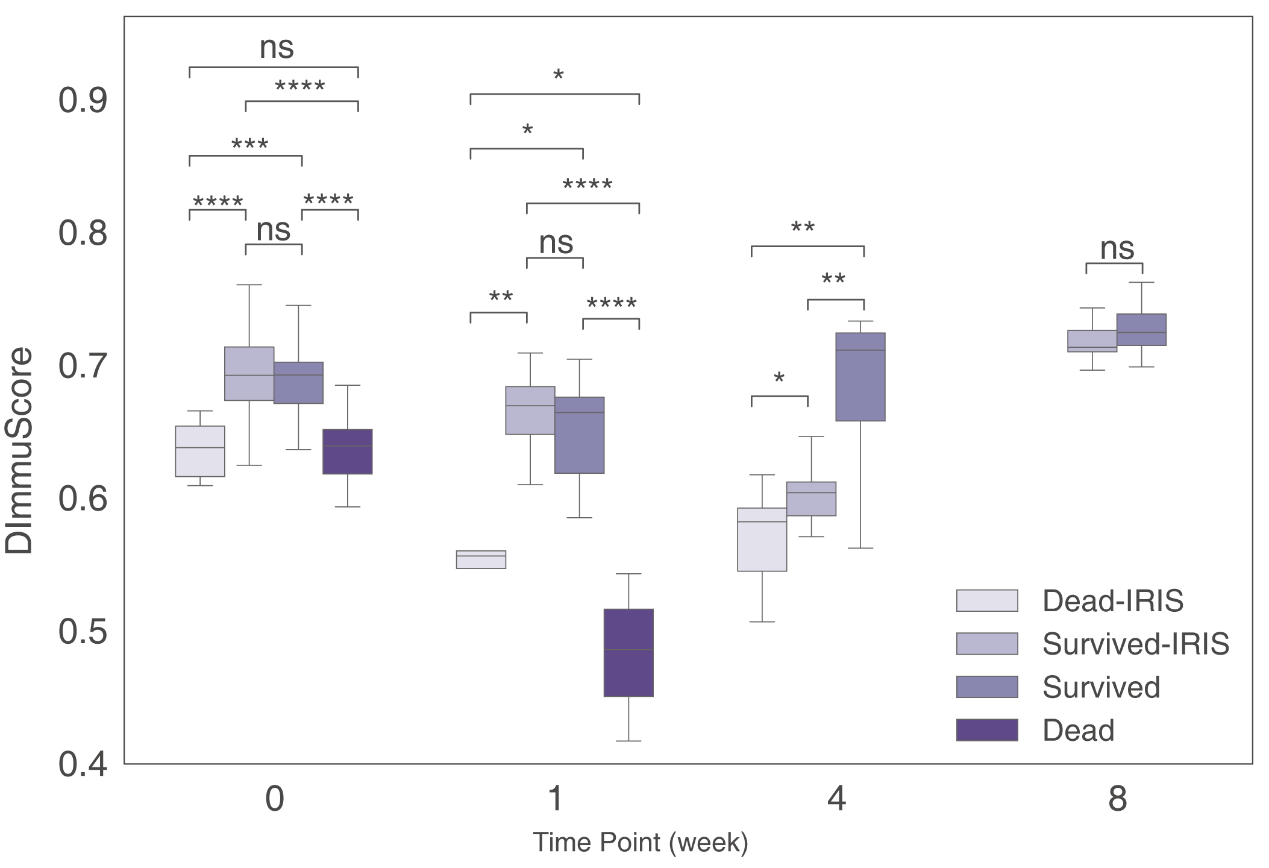


**Supplementary Fig. 9** DImmuScore dynamics in AIDS patients across treatment time

Longitudinal dynamics of DImmuScore in AIDS patients during treatment from week 0 to week 8. (Dead without IRIS, n=33; survived without IRIS, n=70; dead with IRIS, n=22; survived with IRIS, n=68). The significance was assessed by two-side Mann-Whitney U test, ****, p-value ≤ 0.0001; **, p-value ≤ 0.01; ns, p-value < 0.05. IRIS: immune reconstitution inflammatory syndrome.


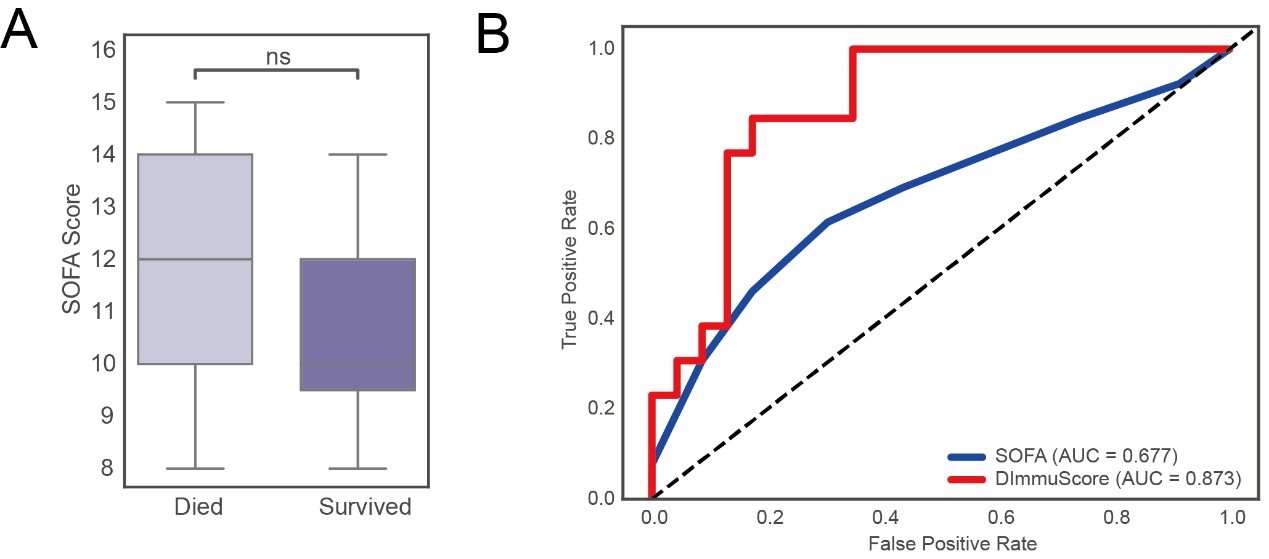


**Supplementary Fig. 10** DImmuScore diagnosis and predict performance compared with SOFA score≥8 in ICU survived/died sepsis patients from PRJNA768419 (n=36; Died=13, Survived=23, PRJNA768419): (**a**) Non significant difference in SOFA scores be-tween died-survived groups (p>0.05, ns); (**b**) ROC curves demonstrating superior predictive accuracy of DImmuScore (AUC=0.873) compared to SOFA (AUC=0.677). ns: no significance. SOFA: sequential organ failure assessment.


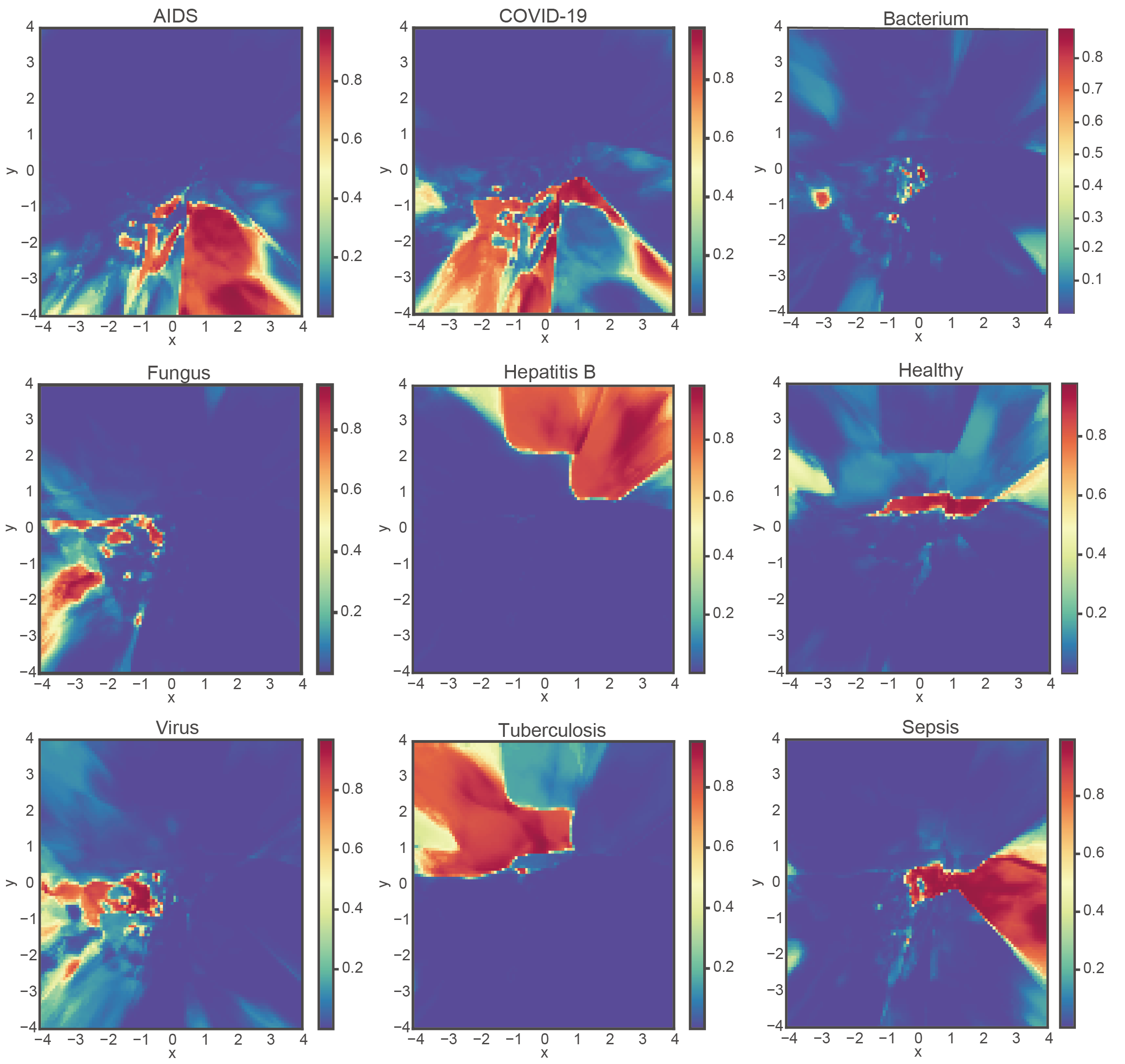
**Supplementary Fig. 11** Heatmaps of predicted average probabilities for nine target classes (AIDS; COVID-19; bacterium; fungus; hepatitis B; healthy; virus; tuberculosis; sepsis) in a multiclass classification task, generated by three ensemble learning models over a latent space grid (x∈[-4,4], y∈[-4,4], 1,000×1,000 sampling resolution).
